# Mechanical Properties of 3D-Printed Nylon-Based Composites Reinforced with Continuous Carbon Fiber: Effect of Reinforcement Layer Distribution

**DOI:** 10.3390/polym18121491

**Published:** 2026-06-13

**Authors:** Boyuan Ding, Jingjing Liu, Mouaz Al Kouzbary, Hanie Nadia Shasmin, Jingang Liu, Shengyan Ge, Noor Azuan Abu Osman

**Affiliations:** 1 School of Mechanical Engineering and Mechanics, Xiangtan University, Xiangtan 411105, China; 202405502301@smail.xtu.edu.cn (B.D.); wellbuild@126.com (J.L.); 2 Engineering Research Center of Complex Track Processing Technology & Equipment, Ministry of Education, Xiangtan University, Xiangtan 411105, China; 3 Key Laboratory of Dynamics and Reliability of Engineering Structures of College of Hunan Province, Xiangtan University, Xiangtan 411105, China; 4 Technology Innovation Center of Theater Technology and Performing Space Equipment Integration, Ministry of Culture and Tourism, Hunan Minghe Cultural Technology Group Co., Ltd., Changsha 410129, China; 13919904000@163.com; 5 Department of Mechanical and Mechatronic Engineering, Curtin University, Miri 98009, Malaysia; mouaz.k@curtin.edu.my; 6 Center for Applied Biomechanics, Department of Biomedical Engineering, Faculty of Engineering, Universiti Malaya, Kuala Lumpur 50603, Malaysia; hanie_nadia@um.edu.my (H.N.S.); azuan@um.edu.my (N.A.A.O.); 7 The Chancellery, Universiti Malaya, Kuala Lumpur 50603, Malaysia

**Keywords:** 3D print, FDM, continuous carbon fiber reinforcement, layer distribution, tensile properties, flexural properties

## Abstract

The application of continuous carbon fiber (CCF) can reinforce the mechanical properties of 3D-printed parts, but the effect of reinforcement layer distribution on composite performance remains unclear. This study investigates the effect of concentrated and separated distributions of CCF layers with different numbers of reinforcement layers. Tensile and flexural tests are conducted in accordance with ASTM D5083 and ASTM D790, respectively. Under the conditions of a solid-filled matrix (Onyx) and 0° CCF deposition, both concentrated and separated CCF layers improve several mechanical properties. Compared with pure Onyx, one-layer CCF increases the tensile modulus by about six times and more than doubles the tensile strength. Increasing the CCF volume leads to further increases in these properties. With concentrated three-layer CCF, the tensile modulus and tensile strength reach 7.153 ± 0.090 GPa and 109.045 ± 5.124 MPa, respectively. For flexural properties, separated two- and three-layer CCFs significantly improve the tangent modulus of elasticity from 0.467 ± 0.106 GPa for pure Onyx to 2.246 ± 0.333 GPa and 3.394 ± 0.081 GPa, respectively. This study also compares the tensile and flexural strength-to-weight ratio of all specimen groups and analyzes the failure mechanisms based on macroscopic fracture appearance. The results can provide guidance for selecting appropriate CCF layer distribution strategies to reinforce composites in different applications.

## 1. Introduction

Three-dimensional (3D) printing, compared with conventional manufacturing methods, shows many advantages, including but not limited to high flexibility of design, ease of fabricating complex structures, and reduction in material waste [1]. Fused deposition modeling (FDM), also known as fused filament fabrication (FFF), is the most common method of 3D printing. In FDM/FFF technology, one or more continuous filaments are heated by the extruder to a semi-liquid state, printed on the base plate layer by layer, and solidified again to form the designed structure [2].

Thermoplastic polymers are the most common materials that can be manufactured by the FDM/FFF method, such as acrylonitrile butadiene styrene (ABS), polylactic acid (PLA, a degradable bioplastic), polyamide (also known as nylon), and polyether ether ketone (PEEK) [3]. For regular 3D printers, various printing parameters, such as layer thickness, infill pattern, infill density, and build orientation, can be adjusted. In more advanced printers with greater process control, additional parameters such as print speed, nozzle temperature, chamber temperature, build plate temperature, and cooling fan speed can also be specified. Many studies have examined how these printing parameters influence the mechanical properties of 3D-printed parts [4].

However, the mechanical properties of thermoplastic polymers, including those reinforced with micro-carbon or glass fibers [5], are still only a fraction of those of engineering metals and cannot satisfy the requirements of some applications. Therefore, the additive manufacturing community is exploring more effective approaches than the addition of microfibers to the matrix material for enhancing the mechanical properties of thermoplastic polymers and composites. Continuous fiber reinforcement shows significant practicability. Carbon fibers, glass fibers and Kevlar fibers are the most common material used for reinforcement [6].

Three methods for continuous fiber reinforcement have been reported in previous studies. First, continuous fibers can be pre-impregnated with a matrix material, such as nylon or resin, to form hybrid composites, which are then manufactured into filaments for 3D printing [7,8]. Second, the most widely studied method at present involves in-nozzle or in situ impregnation of the continuous fiber with the matrix material using a kind of specially designed nozzle [9,10,11]. Third, the continuous fiber and matrix material can be combined during deposition through two separate nozzles. At present, this method is relatively mature, and commercial 3D printers, such as the Mark Two^TM^ desktop 3D printer (Markforged, Watertown, MA, USA), are already available.

Based on the third method, the 3D-printed hybrid composite exhibits an interlayer structure, in which the continuous fiber layers are embedded between layers of matrix material. In such a structure, different types of continuous fibers, including carbon fibers, glass fibers, and Kevlar fibers, serve as the primary determinants of composite performance and lead to distinct mechanical responses [12,13,14,15], including strength and modulus under various loading conditions, such as tension [16], flexure [17], and compression [18]. Because the mechanical properties of these continuous fibers are usually significantly superior to those of the matrix material, researchers also focused on how 3D printing parameters related to the continuous fibers affect the mechanical properties of these hybrid composites.

The fiber volume fraction (or the thickness of continuous fiber layers) is a parameter that readily attracts attention when evaluating various mechanical properties. In general, increasing the amount of continuous fiber is expected to enhance the corresponding mechanical strength and modulus [16], although the extent of improvement is not necessarily proportional [19]. However, the reported conclusions are not fully consistent. For example, the compressive modulus of composites reinforced with continuous glass fiber and Kevlar fiber was found to increase with increasing fiber volume fraction, while the compressive proportional limit did not vary significantly. In contrast, for composites reinforced with continuous carbon fiber (CCF), the compressive proportional limit showed a continuous decline as the fiber volume fraction increased [18]. More complex variations have also been reported. For a nylon-based matrix reinforced with CCF, the compressive modulus first increases and then decreases with increasing fiber volume fraction [18]. Similar non-monotonic behavior has been observed in impact tests. Although the impact strength of continuous-fiber-reinforced samples tends to increase overall with increasing fiber volume fraction, the introduction of a small amount of reinforcing fiber reduces the impact strength of composites relative to that of the matrix material. Only when the fiber content reaches a sufficiently high level does the impact strength begin to improve [20].

Under a given fiber volume fraction, the laying method of continuous fibers has a significant influence on the mechanical properties of the composites. Among the relevant parameters, fiber orientation is particularly important. In tensile [17,19] and compression testing [18,21] of composites reinforced with continuous fiber, it is generally observed that the reinforcing effect is greatest when the fiber direction is parallel to the loading direction, that is, when the fibers are laid at 0°. In contrast, when the fibers are oriented at ±45° or 90°, the reinforcing effect becomes much less significant. Notably, for nylon-based composites reinforced with Kevlar fibers, when the fiber orientation is not parallel to the loading direction, the tensile strength may even fall below that of the matrix material [21]. The influence of fiber orientation on mechanical properties is closely related to the corresponding failure mechanisms. For example, in tensile tests, the failure of specimens reinforced with 0° continuous fiber is primarily governed by fracturing of the continuous fibers, whereas the initial damage of specimens reinforced with ±45° continuous fiber is mainly attributed to debonding between adjacent continuous fiber filaments [17]. In addition to the in-plane fiber direction, spatial fiber orientation also plays an important role. In compression tests, when continuous fibers are deposited in planes perpendicular to the loading direction, an alternating 0°/90° layup provides higher compressive strength than a unidirectional 0° layup [22].

In addition, the layup configuration also involves the distribution of the reinforcement layers. At the same fiber volume fraction, concentrated and separated distributions of continuous-fiber-reinforcement layers can significantly affect the mechanical properties of the composites [17]. This effect is mainly attributed to the complex multi-interface characteristics of 3D-printed continuous fiber composites [23,24,25]. In comparison with traditional manufacturing processes, the interface performance of 3D-printed composites is relatively poor [26,27]. The interfaces between continuous fiber layers, between matrix layers, and between continuous fiber and matrix layers, together with differences in layer orientation, give rise to significantly different failure behaviors [28]. However, in practical applications, one of the primary challenges that engineers face is determining how the continuous fibers should be distributed and how much fibers should be used, that is, the coupling effect between reinforcement layer distribution and continuous fiber volume needs to be quantitatively analyzed. Related studies are still limited.

To fill this gap, this study focuses on the effect of continuous-fiber-reinforcement layer distribution on the mechanical properties of 3D-printed composites while considering variations in continuous fiber volume. The results of this study are expected to support the application of continuous-fiber-reinforced 3D-printed composites in fields requiring both high strength and low weight, such as the structural frame components of powered prostheses [29] and exoskeletons [30].

## 2. Materials and Methods

Standard specimens for tensile and flexural tests were printed by the Mark Two^TM^ desktop 3D printer. The matrix material is Onyx (Markforged, Watertown, MA, USA), which is a kind of fiber-reinforced composite base material that is a micro-carbon-fiber-filled polyamide 6. The reinforced material is CCF (Markforged, Watertown, MA, USA). The mechanical properties of Onyx and carbon fiber measured by the supplier (Markforged) are listed in Table 1.

The tensile and flexural specimens follow the ASTM standards D5083 [31] and D790 [32], respectively. ASTM D5083 was selected instead of ASTM standard D638 [33] because the tensile specimens specified in ASTM D5083 are rectangular prisms, which is consistent with the geometry of the flexural specimens defined in ASTM D790. By contrast, ASTM D638 uses dog-bone-shaped tensile specimens. This selection ensures that, when the reinforcement layer distribution is the same, the volume fraction of CCF in the tensile specimens is consistent with that in the flexural specimens. The tensile specimens are 250 mm in length, 25 mm in width, and 3.375 mm in thickness, whereas the flexural specimens are 66.8 mm in length, 13.5 mm in width, and 3.375 mm in thickness. Because the Mark Two^TM^ desktop 3D printer provides only a single-layer height option of 0.125 mm when CCF is used as reinforcement, the specimen thickness is chosen as an integer multiple of 0.125 mm. Based on the preferred specimen dimensions specified in ASTM D5083 and ASTM D790 and taking into account the convenience of investigating the effect of CCF distribution, 27 layers are selected.

**Figure 1:**
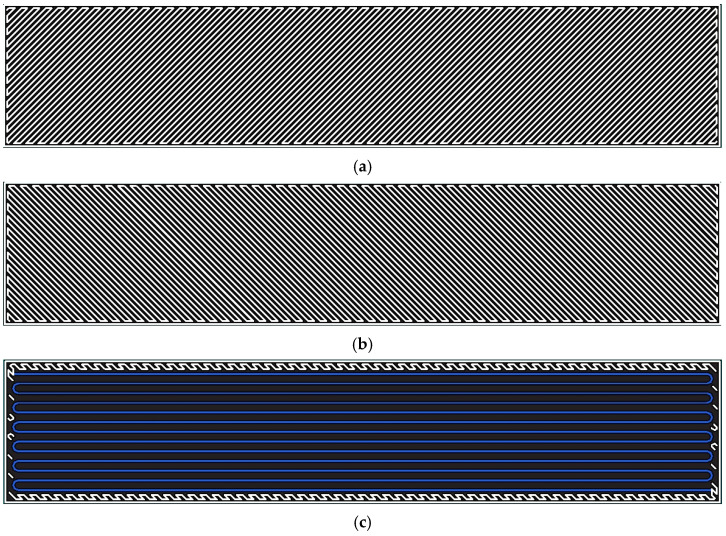
Infill pattern of Onyx (in white) and carbon fiber (in blue) layers in flexural specimens. (**a**) Odd-numbered Onyx layer; (**b**) even-numbered Onyx layer; (**c**) continuous carbon fiber layer.

Besides the abovementioned layer thickness of 0.125 mm of both Onyx and CCF layers, other 3D printing parameters are listed in Table 2. It should be noted that the average volumetric flow rate is estimated by dividing the estimated volume of plastic or fiber by the estimated printing time provided by the Eiger^TM^ platform (Version 3.20.145, Markforged, Watertown, MA, USA), because the deposition speed and volumetric flow rate of the Mark Two^TM^ are not available in official data or online sources. Taking the flexural specimens as an example, under the solid fill pattern, the Onyx layers are deposited alternately at positive and negative 45°, as illustrated in Figure 1a,b. The CCF layers were deposited in the pattern shown in Figure 1c.

**Figure 2:**
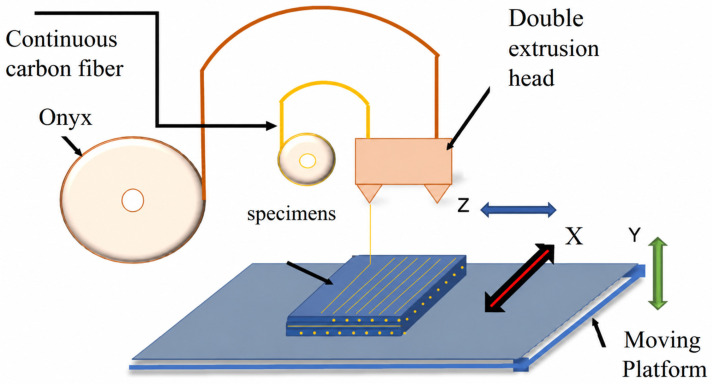
Diagram of 3D printing process using Mark Two^TM^ desktop 3D printer. Diagram sourced from [34].

According to the number and distribution of CCF layers in the specimens, six groups of tensile and flexural specimens were sliced using the Eiger^TM^ platform and then printed. Each specimen was printed separately and measured to ensure that its dimensions met the requirements of the ASTM standards. The diagram of the 3D printing process using the Mark Two^TM^ desktop 3D printer is shown in Figure 2.

The details of each group are listed in Table 3. In the group names, the number indicates the number of CCF layers, while the letters “C” and “S” denote concentrated distribution (in the middle layers of the specimen) and separated distribution (uniformly distributed throughout the 27 layers), respectively. The 1st and the 27th layer represent the bottom and the top surfaces of the specimens, respectively, according to the 3D printing sequence. Tensile and three-point bending tests were conducted using a universal testing machine (Autograph AGS-X Series, Shimadzu, Kyoto, Japan). In the three-point bending tests, the support span was set to 54 mm in accordance with ASTM D790. The corresponding test speeds were selected according to ASTM D5083 and ASTM D790.

## 3. Results

### 3.1. Tensile Properties Results

For each group of tensile specimens, the Eiger^TM^ platform provides the estimated material volume used during printing, and the corresponding weight of the printed part is calculated, as listed in Table 4. In the tensile specimens, a single Onyx layer occupies approximately 0.79 cm^3^, whereas a single CCF layer contains about 0.66 cm^3^ of carbon fiber and 0.11 cm^3^ of Onyx. Since carbon fiber has a higher density (1.4 g/cm^3^) than Onyx (1.2 g/cm^3^), the estimated specimen weight increases with the number of CCF layers.

**Figure 3:**
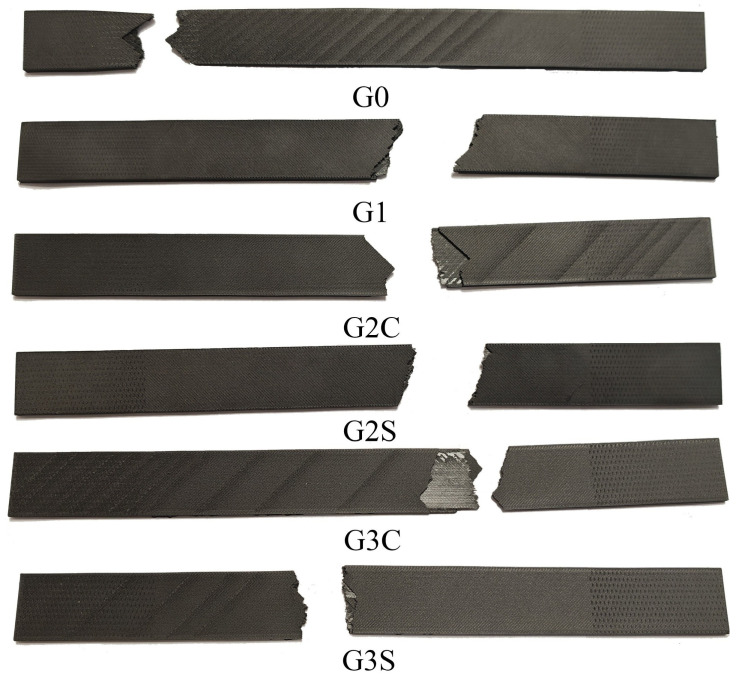
Tensile test results of typical specimens from six groups.

**Figure 4:**
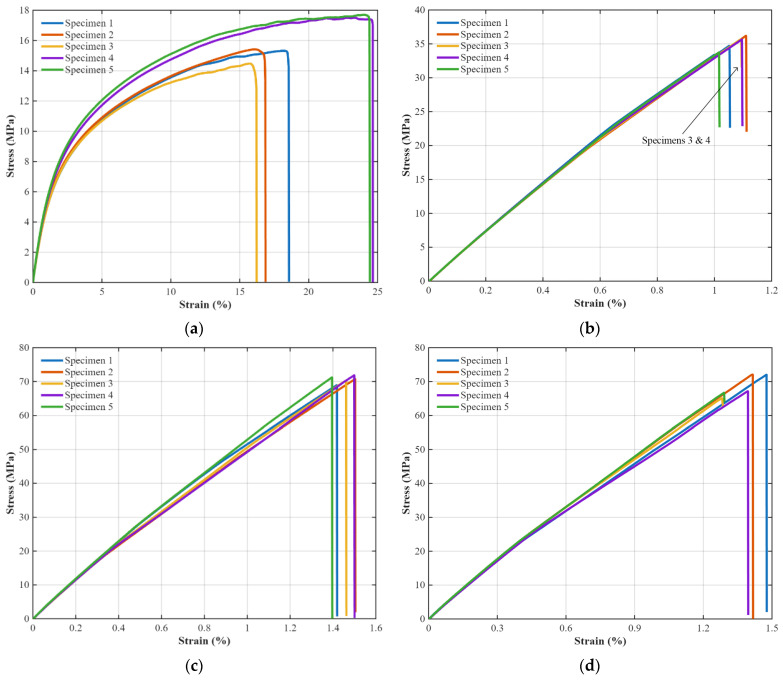

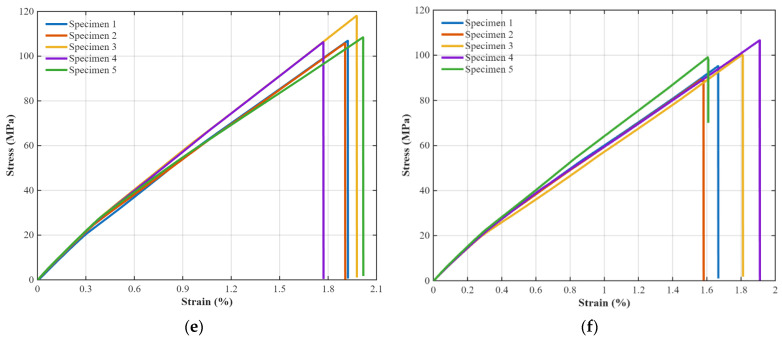
Tensile stress–strain curves of six groups: (**a**) G0; (**b**) G1; (**c**) G2C; (**d**) G2S; (**e**) G3C; (**f**) G3S.

Tensile test results of typical specimens from the six groups and tensile stress–strain curves are illustrated in Figure 3 and Figure 4, respectively. For all groups, a clear initial linear portion of the stress–strain curve can be defined. In this study, the tensile modulus is determined from the strain interval of 0.05% to 0.25%, and the linearity of the fitted region in all groups exceeds 0.999. Beyond this stage, the composites enter a nonlinear deformation region. Among all groups, pure Onyx (G0) shows the most pronounced nonlinear behavior before fracture. In contrast, the composites reinforced with CCF exhibit a second approximately linear region with a lower tensile modulus.

**Figure 5:**
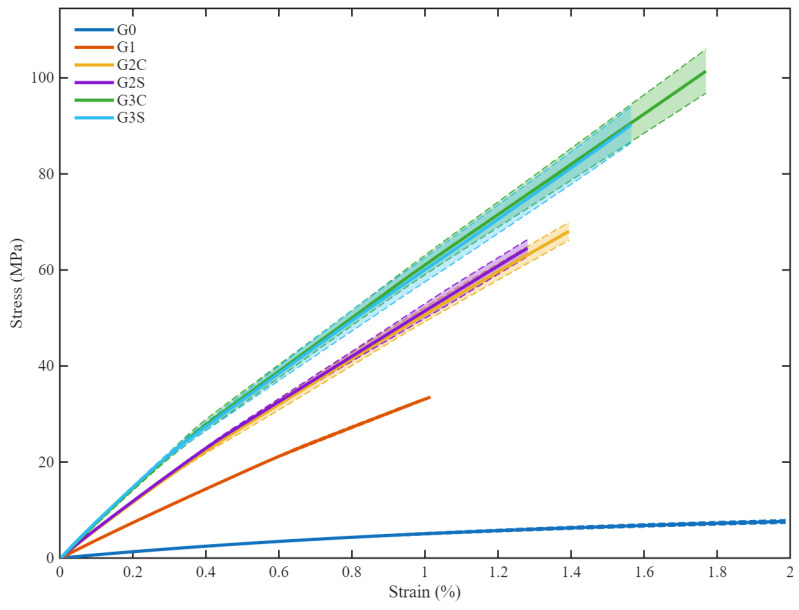
Average tensile stress–strain curves with standard deviation of six groups.

Figure 5 presents the average tensile stress–strain curves with standard deviations for the six groups. In Figure 5, except for G0, the curves of the other five groups are displayed only over the strain range that is meaningful for all five specimens, following the presentation method used in the literature [35]. As a basis for comparison, the curve of G0 is presented up to a strain of 2%. The dispersion degree of the results among the six groups can be categorized into three levels. G0 and G1 exhibit relatively low dispersion degree, with G1 even showing lower dispersion than G0. As the CCF volume increases, the dispersion degree of the results also increases, whereas the influence of reinforcement layer distribution on the dispersion degree remains limited.

For all groups, no apparent yield point is observed in the tensile stress–strain curves. Moreover, the 0.2% strain offset method is not applicable to composites reinforced with CCF, because the intersection point does not occur before fracture. Therefore, a deviation-from-linearity criterion is adopted in this study. The initial linear portion of the stress–strain curves is fitted by linear regression over the strain interval of 0.05–0.25%. Then, the onset of nonlinear deformation is defined as the first point at which the measured stress deviates from the extrapolated linear fit by more than 5% over consecutive data points. For each tensile specimen (five specimens per group), the fitted line for the initial linear region and the identified onset point of nonlinear deformation are provided in Supplementary Material Figure S1.

The tensile properties, including the tensile stress and strain at the onset of nonlinear deformation, are summarized in Table 5 as the form of mean values (standard deviations). Pure Onyx (G0) shows high extensibility, with a tensile strain at break of 19.443 ± 3.941%, although the tensile strain at the onset of nonlinear deformation is only 0.403 ± 0.005%. Reinforcement with CCF significantly reduces the extensibility of the composites but increases the tensile strain at the onset of nonlinear deformation. An increase in the number of CCF layers leads to a lower tensile strain at the onset of nonlinear deformation but a higher tensile strain at break. For the same number of CCF layers, the separated distribution yields lower tensile strain at the onset of nonlinear deformation and lower tensile strain at break than the corresponding concentrated distribution.

**Figure 6:**
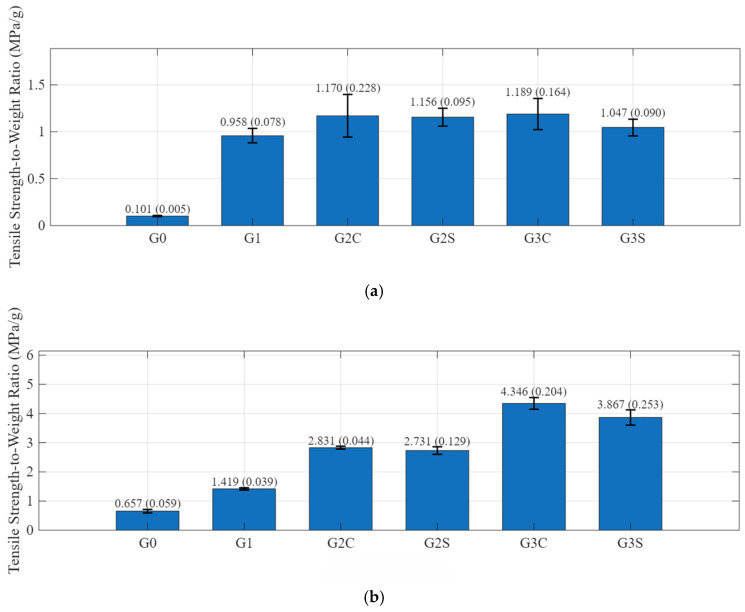
Tensile strength-to-weight ratio of six groups: (**a**) at onset of nonlinear deformation; (**b**) at break.

With regard to tensile strength, in addition to directly comparing the tensile stress at the onset of nonlinear deformation and at break among different groups, the tensile stress per unit mass, i.e., the tensile strength-to-weight ratio, is also calculated to assess the reinforcing efficiency of CCF, as shown in Figure 6.

For the tensile stress at onset of nonlinear deformation, the introduction of one layer of CCF (G1) increases the value to more than 9.5 times that of pure Onyx (G0). At the same time, the tensile strength-to-weight ratio in the linear region also improves by more than 9.5 times. Further increasing the number of CCF layers to two or three yields results in only a limited additional improvement in the tensile stress at the onset of nonlinear deformation, with the increase remaining within 25% relative to the one-layer CCF specimen (G1). Notably, G3S exhibits a lower tensile stress at the onset of nonlinear deformation than both G2C and G2S, whereas G3C achieves the highest tensile strength-to-weight ratio.

For the tensile stress at break, G1 exhibits a value more than twice that of G0. G2C and G3C, which contain two and three times the number of CCF layers in G1, respectively, also show a corresponding increase in tensile stress at break relative to G1. By contrast, G2S and G3S exhibit lower values than G2C and G3C, respectively. A similar trend is also observed in the tensile strength-to-weight ratio at break. Among all the investigated groups, G3C achieves the highest tensile strength-to-weight ratio, with a value of 4.207.

### 3.2. Flexural Properties Results

For each group of flexural specimens, the estimated weight is also calculated, as listed in Table 6. In the flexural specimens, a single Onyx layer occupies approximately 0.11 cm^3^, whereas a single CCF layer contains about 0.09 cm^3^ of carbon fiber and 0.02 cm^3^ of Onyx.

**Figure 7:**
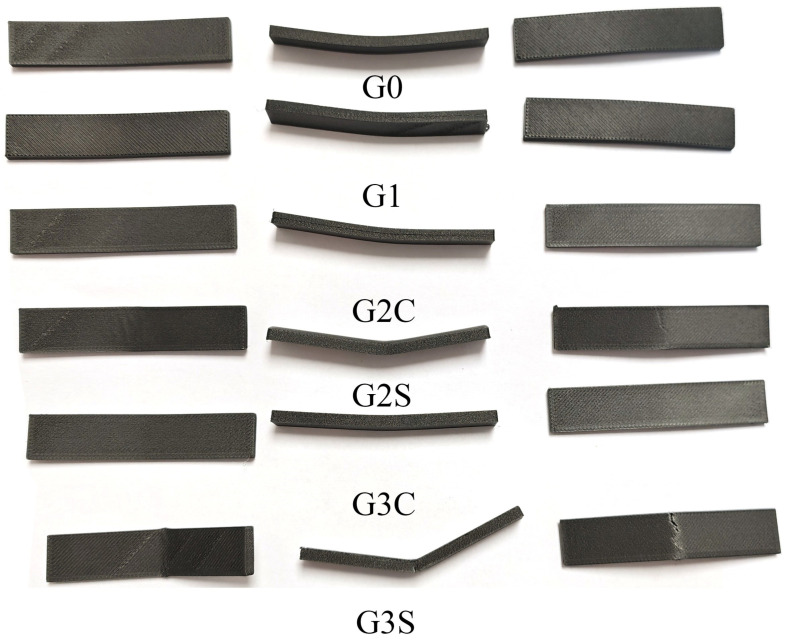
Flexural test results of typical specimens from six groups.

**Figure 8:**
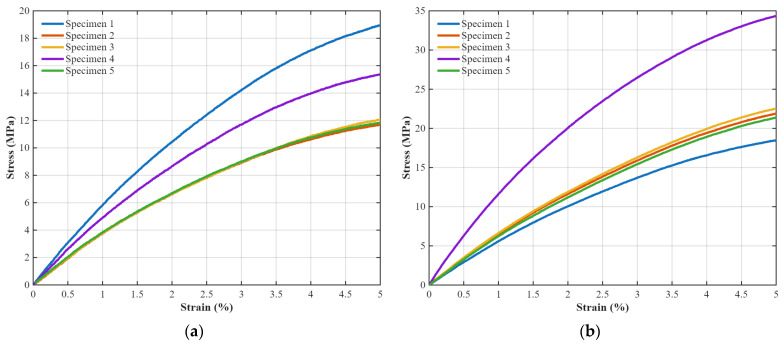

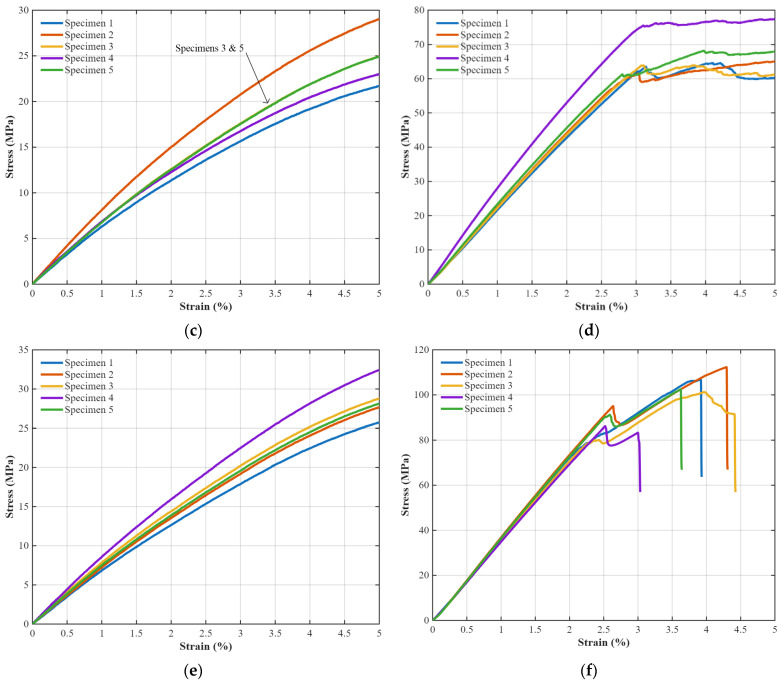
Flexural stress–strain curves of six groups: (**a**) G0; (**b**) G1; (**c**) G2C; (**d**) G2S; (**e**) G3C; (**f**) G3S.

The flexural test results of typical specimens from the six groups and flexural stress–strain curves are illustrated in Figure 7 and Figure 8, respectively. According to ASTM D790, the flexural response is reported up to a strain of 5%. The complete flexural stress–strain curves up to the complete failure in flexural load-carrying capacity of the specimens are provided in Supplementary Material Figure S2. Similar to the tensile stress–strain curves, a distinct initial linear region can be identified for all groups. In this study, the tangent modulus of elasticity is determined from the strain interval of 0.05% to 0.25%, and the linearity of the fitted region in all groups exceeds 0.998. For specimens of pure Onyx (G0) and those with CCF concentrated in the center (G1, G2C, and G3C), no fracture occurs before the strain reaches 5%. In these four groups, the composites enter a nonlinear deformation region after the initial linear stage.

**Figure 9:**
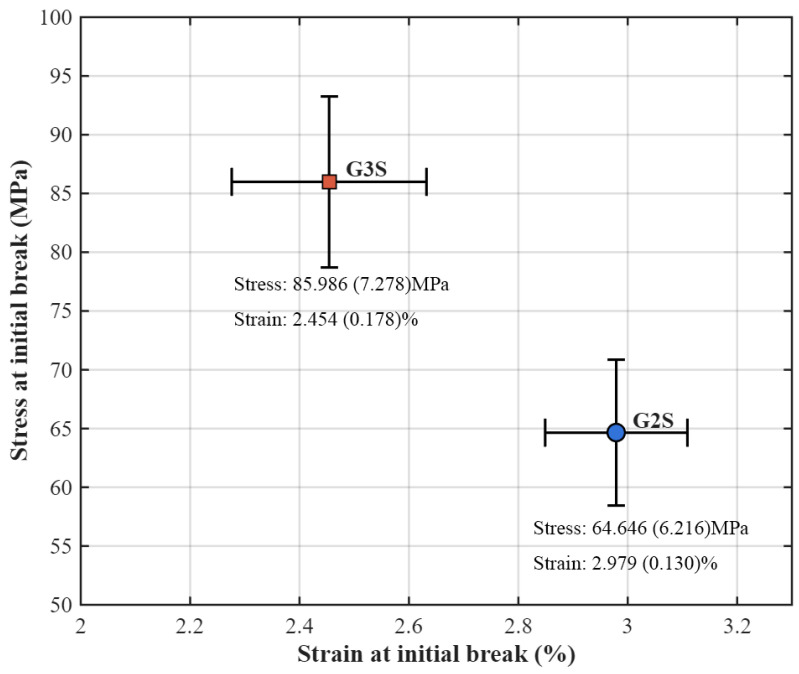
Flexural stress and strain with standard deviation at initial break for G2S and G3S.

For G2S and G3S, the initial linear region is significantly shorter than that of the other four groups, whereas the modulus in the nonlinear deformation region does not show an obvious change. An initial break is observed in G2S at a strain of 2.979 ± 0.130%. This initial break does not cause failure of the flexural load-carrying capacity. After the initial break, the flexural stress in the G2S specimens fluctuates and continues to rise. A similar initial break is observed in G3S at a strain of 2.454 ± 0.178%. Figure 9 presents the flexural stress and strain at the initial fracture for G2S and G3S, together with their standard deviations. In G3S, the flexural stress drops by approximately 5–8% immediately after the initial break and then recovers or even increases further. When complete fracture occurs, the flexural stress decreases rapidly by more than 20%.

Using the same approach and criterion as those applied in the tensile analysis, the initial linear portion of the flexural stress–strain curves is fitted by linear regression, and the onset of nonlinear deformation is defined. The detailed results for each flexural specimen (five specimens per group) are provided in Supplementary Material Figure S3.

**Figure 10:**
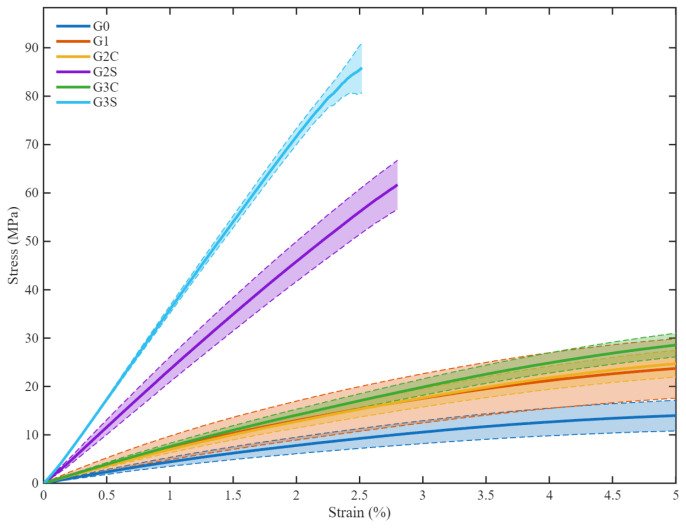
Average flexural stress–strain curves with standard deviation of six groups.

Figure 10 presents the average flexural stress–strain curves with standard deviations for the six groups. The curves of G0, G1, G2C, and G3C are shown up to a strain of 5%. Since fracture occurs in G2S and G3S before 5% strain, the curves of these two groups, similar to those in Figure 5, are displayed only over the strain range that is meaningful for all five specimens. Figure 10 shows that only G3S exhibits relatively low dispersion degree. In addition, at the same strain level, the stress values of G1, G2C, and G3C are only slightly higher than that of G0.

The flexural properties, including the flexural stress and strain at the onset of nonlinear deformation, are summarized in Table 7 as mean values with standard deviations. Pure Onyx (G0) exhibits a low tangent modulus of elasticity (only 0.467 GPa), whereas specimens reinforced with CCF in a concentrated distribution (G1, G2C, and G3C) improve this value by no more than 1.7 times. By contrast, G2S and G3S significantly increase the tangent modulus of elasticity, reaching more than 4.8-fold and 7.2-fold that of G0, respectively. Compared with the tensile behavior, the initial linear deformation region under flexural loading is larger for G0, G1, G2C, and G3C, where the flexural strain of linear deformation can exceed 0.8%. However, the separated distribution of reinforced fibers in G3S markedly shortens this linear region.

Taking the flexural stress at the onset of nonlinear deformation of pure Onyx (G0), 4.194 MPa, as a reference, one to three layers of CCF in a concentrated distribution (G1, G2C, and G3C) increase this value by over 40% to 126%. G3S shows a lower value for this property than pure Onyx (G0).

Notably, for G2S, both the flexural stress and strain at the onset of nonlinear deformation exhibit a high degree of dispersion, such that the standard deviations are even greater than the corresponding mean values. Within this group, two specimens show a high degree of linearity, with the strain at the onset of nonlinearity exceeding 1.7% and 3.1%. If these two specimens are excluded, the flexural stress and strain at the onset of nonlinear deformation for G2S are 7.383 ± 0.794 MPa and 0.343 ± 0.025%, respectively.

**Figure 11:**
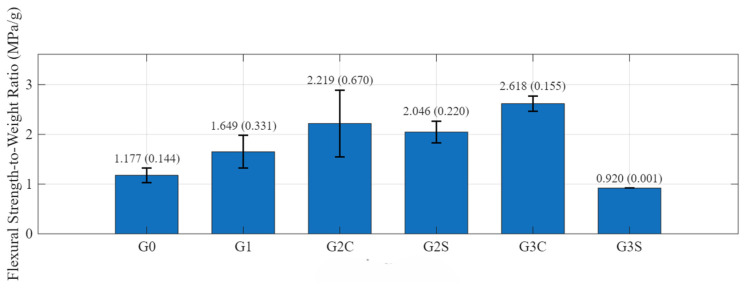
Flexural strength-to-weight ratio of six groups at onset of nonlinear deformation.

Referring to the estimated weight of the flexural specimens in Table 5, the flexural stress per unit mass, i.e., the flexural strength-to-weight ratio, is calculated and shown in Figure 11. Since fracture does not occur for most groups, the comparison is limited to the performance at the onset of nonlinear deformation. It can be seen that the flexural strength-to-weight ratio shows nearly the same trend as the flexural stress at the onset of nonlinear deformation, although the extent of improvement differs.

## 4. Discussion

**Figure 12:**
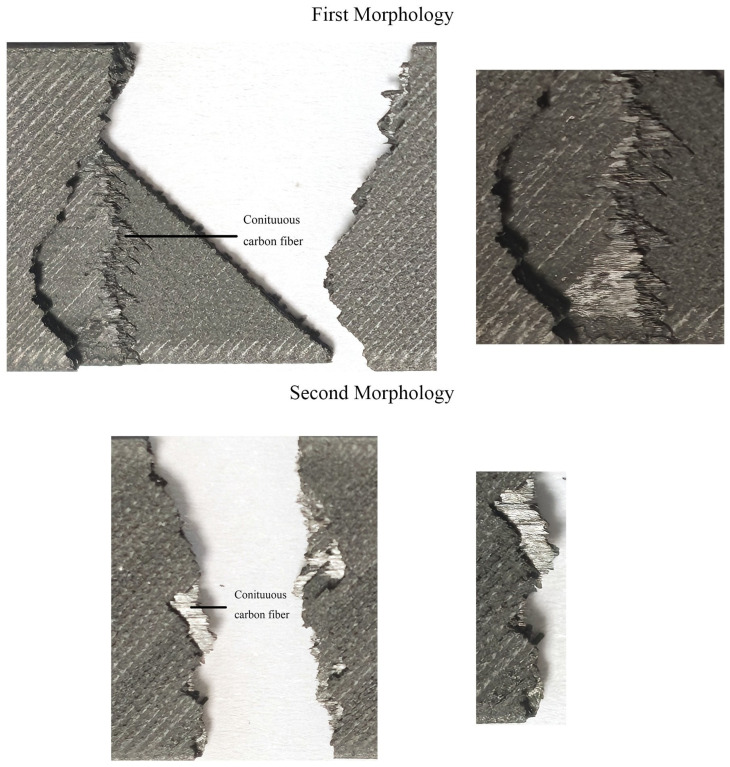
Details for two typical fracture morphologies.

Although the present study does not examine the tensile and flexural specimens at the microscale, the results still contribute to the understanding of the failure modes of 3D-printed parts reinforced with CCF based on the macroscopic fracture appearance of the specimens. For the tensile specimens shown in Figure 3, two typical fracture morphologies can be identified. The first morphology is characterized by a pronounced difference between the fracture appearances of the Onyx layers and the CCF layers. Specifically, the length span of the fracture region of the Onyx is large, whereas that of the CCF is much shorter. A large area of Onyx is delaminated from the CCF layers. This morphology is mainly observed in the G2C and G3C specimens. The second morphology shows a limited difference between the fracture appearances of the Onyx and CCF layers. The fracture regions of both materials extend over similar lengths, and only a small amount of Onyx is delaminated from the CCF layers. This morphology is mainly observed in the G2S and G3S specimens. Although G0 contains no CCF reinforcement, the fracture appearance of its Onyx region is more similar to the first morphology, whereas that of G1 is closer to the second. The details of the first and second morphologies in representative specimens are illustrated in Figure 12.

The different failure morphologies can be interpreted at multiple levels. First, two underlying conditions should be considered: the elongation of Onyx is much higher than that of CCF, and in all groups, Onyx is deposited at positive and negative 45°, as shown in Figure 1. Under tensile loading, the fracture of Onyx may occur in two forms: cracking perpendicular to the filament direction and separation between adjacent filaments. At the macroscopic level, this leads to a triangular fracture region with a relatively large length span, as clearly shown by the fracture region of G0. The Onyx layers in G2C and G3C also show the same feature, suggesting that failure in these specimens likely initiates with fracture of the central CCF layer, followed by delamination of the outer Onyx from the CCF layer, and ultimately fracture of the Onyx.

Second, in the macroscopic fracture morphologies described above, delamination is observed only between the Onyx and CCF layers. No obvious delamination is found between adjacent Onyx layers or between adjacent CCF layers in the macroscopic failure region. This indicates that the interlayer bonding strength of the Onyx–Onyx interface is higher than that of the Onyx–CCF interface. A similar conclusion can also be inferred from the theoretical analysis [36], but direct experimental evidence has not yet been reported.

Third, when the CCF is deposited in a separated distribution, delamination of Onyx from the CCF layers becomes less obvious. One possible reason is that, in the separated distribution, only two types of interlayer interfaces are involved, namely the Onyx–Onyx interface and the Onyx–CCF interface. Although the bonding strength of the former is higher than the counterpart of the latter, the difference may not be large enough to cause pronounced delamination. Instead, when the CCF layer fractures first, damage is transferred to the adjacent Onyx layers through the Onyx–CCF interface, which promotes fracture propagation. As a result, the Onyx layers fracture, followed by fracture of the CCF layers, and the specimen fails without obvious macroscopic delamination.

Fourth, a combined consideration of the second and third levels suggests that the Onyx–CCF interface is more susceptible to delamination when it is adjacent to a CCF–CCF interface than when it is adjacent to an Onyx–Onyx interface. This may indicate that the interlayer bonding strength of the CCF–CCF interface is substantially greater than the counterparts of the other two interlayer interface types, which is consistent with the results from a previous study [28].

**Figure 13:**
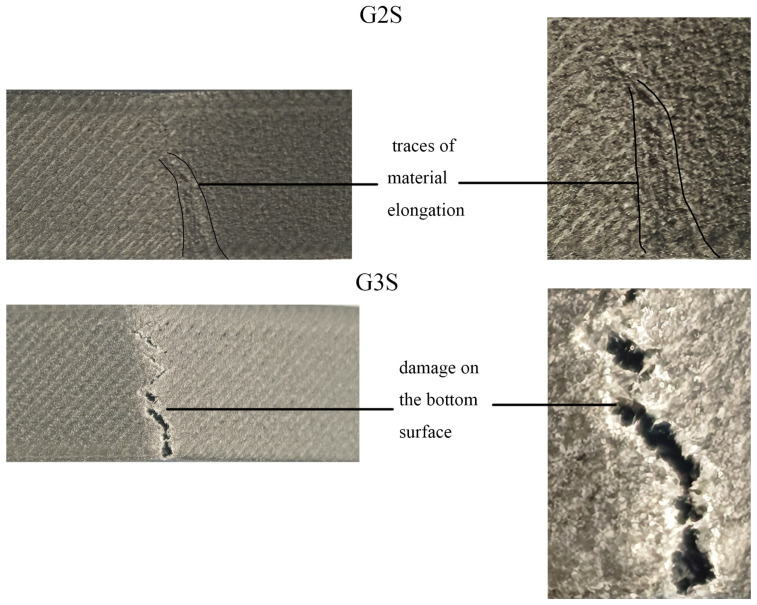
Details for bottom surface change in G2S and G3S.

For the flexural specimens shown in Figure 5, even after the pure Onyx specimens (G0) and the specimens with CCF concentrated in the center (G1, G2C, and G3C) had completely lost their flexural load-carrying capacity, no obvious surface changes were observed on the top, bottom, or side surfaces. Moreover, the bending deformation was largely recoverable after unloading. In contrast, for G2S, the recovery after unloading was significantly reduced and was observed on the bottom surface. For G3S, obvious damage was observed on the bottom and side surfaces, along with creases on the top surface. After unloading, the bending deformation was hardly recoverable. The details of bottom surface of G2S and G3S are illustrated in Figure 13. The surface traces and damage observed in G2S and G3S may be related to the fact that fracture of the CCF occurred first, which then caused damage in the adjacent Onyx layers through the Onyx–CCF interface, thereby promoting fracture propagation. However, these inferences regarding the interfacial strength of different materials and the fracture propagation mechanism require further verification through specially designed specimens.

On the aspect of mechanical properties, it is worth noting that pure Onyx specimens (G0), even with a solid fill pattern, differ significantly from the raw material. First, no distinct yield point is observed in the tensile stress–strain curves of the 3D-printed Onyx parts. Second, the tensile modulus and tangent modulus of elasticity reach less than 27% and 16% of the nominal material properties, respectively. Third, the tensile stress at break was only 43.47% of that of the material itself. Even after reinforcement with CCF layers, the tensile stress at break merely approaches the nominal value of Onyx. The only property that remains relatively close is the tensile strain at break, which reached 77.77% of the nominal material value. Previous studies [14,37,38] have also reported different mechanical properties for pure Onyx under various printing parameters. In those studies, the tensile stress at break is close to the nominal value of Onyx, approximately 37 MPa, whereas the tensile strain at break is only 0.25–0.8%. These results suggest that, in practical applications, the infill strategy and printing parameters should be carefully investigated, and appropriate trade-offs between strength and extensibility should be made according to specific requirements.

For the tensile stress at break, i.e., the tensile strength, the values show a good linear relationship with the number of CCF layers. This suggests that the contribution of the matrix to tensile strength becomes limited once the structure is reinforced with one or more CCF layers. Meanwhile, the separated distribution of CCF yields slightly lower values than the concentrated distribution. This observation further supports the above analysis that the interlayer bonding strength of the CCF–CCF interface is the greatest among the three types of interfaces and contributes to the tensile strength of the overall structure [39].

Finally, the primary focus of this study is the number and distribution of CCF layers. In most practical applications involving supporting structures, the nonlinear deformation is regarded as structural failure. Accordingly, the mechanical properties at the onset of nonlinear deformation are taken as the main basis for analysis.

With respect to tensile performance, the reinforcing efficiency gradually decreases as the number of CCF layers increases from one to three. Relative to the pure matrix material, a single layer of CCF reinforcement increases the tensile stress at the onset of nonlinear deformation by more than 21 MPa. However, under a concentrated distribution, the addition of a second layer and a third layer yields only limited further increases of approximately 5.43 MPa and 0.65 MPa, respectively. This implies that the reinforcing effect of additional CCF layers beyond three layers is highly limited and may not lead to further effective enhancement. The reinforcing effect under a separated distribution of CCF is even weaker, that is, although the volume of CCF remains the same, the separated distribution produces a slightly lower tensile stress at the onset of nonlinear deformation than the concentrated distribution. In addition, as abovementioned, increasing the amount of CCF reduces the strain range of the linear deformation region, thereby imposing stricter constraints on practical applications.

Regarding the tensile strength-to-weight ratio, the reinforcement of CCF improves the tensile load-carrying capacity per unit mass relative to the pure matrix material. This suggests considerable potential for the application of such materials in lightweight complex structures, while also exploiting the key advantage of 3D printing in fabricating complex geometries. Although the density of CCF is higher than that of the matrix material, increasing the amount of CCF still enhances the tensile load-carrying efficiency at the onset of nonlinear deformation, albeit to a limited extent. Under extreme loading conditions approaching fracture, increasing the amount of CCF provides a supporting efficiency that is approximately proportional to the amount of CCF.

From the perspective of flexural performance, the separated distribution of CCF is highly effective in increasing the tangent modulus of elasticity. However, for the same amount of CCF, it results in lower flexural strength and a shorter linear deformation region than the concentrated distribution. More importantly, when the CCF layers are positioned close to the specimen surface, they are more likely to fracture under flexural loading. The closer they are to the surface, the earlier the fracture occurs. Nevertheless, this initial fracture does not correspond to complete loss of flexural load-carrying capacity.

The evaluation of flexural load-carrying efficiency also focuses primarily on the onset of nonlinear deformation. Compared with the matrix material, a single layer of CCF reinforcement does not enhance flexural load-carrying efficiency as markedly as it enhances tensile load-carrying efficiency. However, as the number of concentrated CCF layers increases, effective improvement can still be maintained. In contrast, the separated distribution yields lower flexural load-carrying efficiency than the same amount of CCF in a concentrated distribution. Notably, when the CCF layers are positioned too close to the surface, the flexural load-carrying efficiency of the composite may even worsen below that of the matrix material.

**Figure 14:**
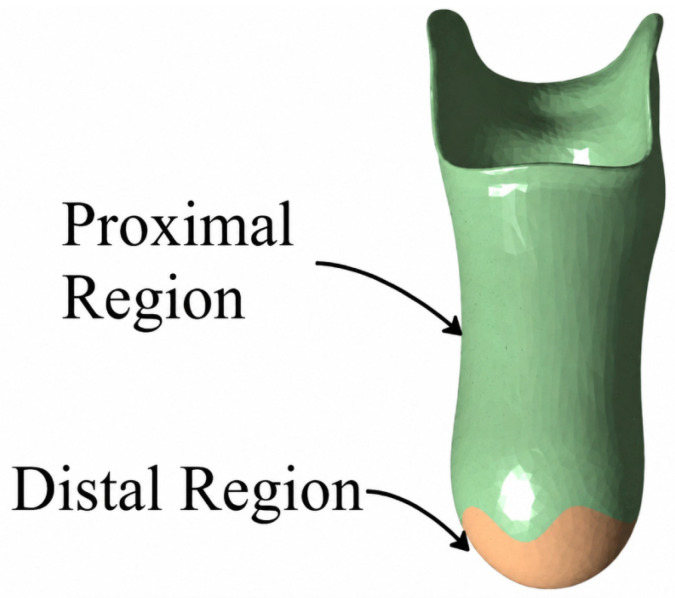
Schematic diagram of distal and proximal segmentations of prosthetic socket.

For applications in systems of powered prostheses and powered exoskeletons, CCF reinforcement can be regarded as a feasible manufacturing strategy for key structural components because of the strong demand for lightweight design. For instance, components directly related to human wear, the proximal region of the prosthetic socket, and other flexible connection structures generally require relatively low bending stiffness together with high strength. In these cases, a concentrated distribution of multiple CCF layers may be selected to better satisfy comfort requirements during wear. In contrast, the main structural shell, the supporting structure of the transmission mechanism, and the rigid connections between different components may be better reinforced by a separated distribution of multiple CCF layers. Such a configuration helps maintain higher geometric accuracy and prevents deterioration in the assembly precision caused by deformation or transmission jamming. The two distributions may also be combined in a functionally graded manner. For example, in the prosthetic socket shown in Figure 14, concentrated and separated distributions may be applied to the proximal and distal regions, respectively [40], in order to simultaneously achieve wearing comfort and geometric stability of the adapter connection.

## 5. Conclusions

This study investigates the effect of reinforcement layer distribution on the mechanical properties of 3D-printed nylon-based composites reinforced with CCF. The variables among the different specimen groups include the number of CCF reinforcement layers, ranging from one to three, and their distribution within the specimens, namely concentrated and separated distributions. The results of the tensile and flexural tests demonstrate that reinforcement layer distribution significantly affects the mechanical properties, including the tensile/flexural modulus, the tensile/flexural stress and strain at different deformation stages, and the tensile/flexural strength-to-weight ratio. Under tensile loading, one-layer CCF increases the tensile modulus by about six times and more than doubles the tensile strength. With concentrated three-layer CCF, the tensile modulus and tensile strength further increase to 7.153 ± 0.090 GPa and 109.045 ± 5.124 MPa, respectively. Under flexural loading, separated two- and three-layer CCFs significantly improve the tangent modulus of elasticity from 0.467 ± 0.106 GPa for pure Onyx to 2.246 ± 0.333 GPa and 3.394 ± 0.081 GPa, respectively. Besides the trends of the indicators of mechanical properties, this study also analyzes the failure mechanisms based on the macroscopic fracture appearance of the specimens. Finally, with powered prostheses and powered exoskeletons taken as representative application scenarios, the potential application prospects of these composites are discussed.

## Figures and Tables

**Table 1:** Mechanical properties of Onyx and carbon fiber.

Mechanical Properties	Onyx	Carbon Fiber
Tensile Modulus (GPa)	2.4	60
Tensile Stress at Yield (MPa)	40	N/A
Tensile Stress at Break/Tensile Strength (MPa)	37	800
Tensile Strain at Break (%)	25	1.5
Flexural Strength (MPa)	71	540
Flexural Modulus (GPa)	3.0	51
Flexural Strain at Break (%)	N/A	1.2

**Table 2:** 3D printing parameters of tensile and flexural specimens.

Matrix Material (Onyx)		Continuous Carbon Fiber	
Nozzle diameter	0.4 mm	Nozzle diameter	0.9 mm
Heat temperature	265 °C	Heat temperature	270 °C
Estimated average volumetric flow rate	0.129 cm^3^/min	Estimated average volumetric flow rate	0.054 cm^3^/min
Fill pattern (fill density)	Solid fill (100%)	Fill type	Isotropic fiber
Wall layer	1 layer	Concentric rings	0
Wall thickness	0.4 mm	Start rotation %	0
		Angles	0

**Table 3:** Layer distribution of six groups.

Groups	Layers of Onyx	Layers of Continuous Carbon Fiber
G0	1st to 27th	N/A
G1	1st to 13th and 15th to 27th	14th
G2C	1st to 13th and 16th to 27th	14th and 15th
G2S	1st to 8th, 10th to 18th and 20th to 27th	9th and 19th
G3C	1st to 12th and 16th to 27th	13th to 15th
G3S	1st to 6th, 8th to 13th, 15th to 20th and 22nd to 27th	7th, 14th and 21st

**Table 4:** Estimated parameters of tensile specimens obtained from Eiger^TM^ (Markforged).

Groups	Estimated Fiber Volume (cm^3^)	Estimated Plastic Volume (cm^3^)	Estimated Weight (g)
G0	0	21.33	25.596
G1	0.66	20.65	25.704
G2C	1.32	19.97	25.812
G2S	1.32	19.97	25.812
G3C	1.98	19.29	25.920
G3S	1.98	19.29	25.920

**Table 5:** Tensile properties of six groups.

Groups	Tensile Modulus (GPa)	Tensile Stress at Onset of Nonlinear Deformation (MPa)	Tensile Strain at Onset of Nonlinear Deformation (%)	Tensile Stress at Break (MPa)	Tensile Strain at Break (%)
G0	0.638 (0.027)	2.484 (0.134)	0.403 (0.005)	16.084 (1.437)	19.443 (3.941)
G1	3.653 (0.017)	23.748 (1.940)	0.683 (0.055)	35.188 (0.975)	1.073 (0.039)
G2C	5.658 (0.077)	29.182 (5.674)	0.537 (0.098)	70.599 (1.101)	1.455 (0.048)
G2S	5.766 (0.071)	29.033 (2.396)	0.525 (0.043)	68.609 (3.230)	1.371 (0.083)
G3C	7.153 (0.090)	29.833 (4.108)	0.436 (0.053)	109.045 (5.124)	1.918 (0.094)
G3S	7.178 (0.131)	26.543 (2.275)	0.384 (0.030)	98.059 (6.422)	1.710 (0.144)

**Table 6:** Estimated parameters of flexural specimens obtained from Eiger^TM^ (Markforged).

Groups	Estimated Fiber Volume (cm^3^)	Estimated Plastic Volume (cm^3^)	Estimated Weight (g)
G0	0	2.97	3.564
G1	0.11	2.86	3.586
G2C	0.22	2.75	3.608
G2S	0.22	2.75	3.608
G3C	0.33	2.64	3.630
G3S	0.33	2.64	3.630

**Table 7:** Flexural properties of six groups.

Groups	Tangent Modulus of Elasticity (GPa)	Flexural Stress at Onset of Nonlinear Deformation (MPa)	Flexural Strain at Onset of Nonlinear Deformation (%)	Flexural Stress at 5% Strain (MPa)
G0	0.467 (0.106)	4.194 (0.512)	0.978 (0.222)	13.994 (3.167)
G1	0.791 (0.298)	5.914 (1.187)	0.846 (0.272)	23.723 (6.140)
G2C	0.723 (0.069)	8.005 (2.418)	1.163 (0.336)	24.724 (2.779)
G2S	2.246 (0.333)	26.614 (26.864)	1.193 (1.260)	N/A
G3C	0.783 (0.077)	9.503 (0.561)	1.291 (0.177)	28.564 (2.461)
G3S	3.394 (0.081)	3.338 (0.005)	0.528 (0.091)	N/A

## Data Availability

The raw data supporting the conclusions of this article will be made available by the authors on request.

## Supplementary Materials

The following supporting information can be downloaded at: https://www.mdpi.com/article/10.3390/polym18121491/s1, Figure S1: Fitted line for the initial linear region and onset point of nonlinear deformation of each tensile specimen: (a) G0; (b) G1; (c) G2C; (d) G2S; (e) G3C; (f) G3S; Figure S2: Complete flexural stress–strain curves of flexural specimens: (a) G0; (b) G1; (c) G2C; (d) G2S; (e) G3C; (f) G3S; Figure S3: Fitted line for the initial linear region and onset point of nonlinear deformation of each flexural specimen: (a) G0; (b) G1; (c) G2C; (d) G2S; (e) G3C; (f) G3S.

## Abbreviation

The following abbreviation is used in this manuscript:

CCF	Continuous carbon fiber
