# Supplementary material for: Mechanical Properties of 3D-Printed Nylon-Based Composites Reinforced with Continuous Carbon Fiber: Effect of Reinforcement Layer Distribution
Source: Polymers (Basel). 2026 Jun 13;18(12):1491. doi: 10.3390/polym18121491 (PMC13307125; doi:10.3390/polym18121491)
Supplement: Supplementary file 1 [file polymers-18-01491-s001.zip › polymers-4349107-supplementary.pdf]

Mechanical Properties of 3D-Printed Nylon-Based Composites Reinforced with Continuous Carbon Fiber: Effect of Reinforcement Layer Distribution

Boyuan Ding [1], Jingjing Liu [1,2,3,4,*], Mouaz Al Kouzbary [5], Hanie Nadia Shasmin [6], Jingang Liu [1], Shengyan Ge [4], and Noor Azuan Abu Osman [6,7]

1 School of Mechanical Engineering and Mechanics, Xiangtan University, Xiangtan, Hunan 411105, China;
2 Engineering Research Center of Complex Track Processing Technology & Equipment, Ministry of Education, Xiangtan University, Xiangtan 411105, China;
3 Key Laboratory of Dynamics and Reliability of Engineering Structures of College of Hunan Province, Xiangtan University, Xiangtan 411105, China;
4 Technology Innovation Center of Theater Technology and Performing Space Equipment Integration, Ministry of Culture and Tourism, Hunan Minghe Cultural Technology Group Co., Ltd., Changsha, Hunan 410129, China;
5 Department of mechanical and mechatronic engineering, Curtin University, Miri, Sarawak 98009, Malaysia;
6 Center for Applied Biomechanics, Department of Biomedical Engineering, Faculty of Engineering, Universiti Malaya, Kuala Lumpur 50603, Malaysia
7 The Chancellery, Universiti Malaya, Kuala Lumpur 50603, Malaysia
* Correspondence: liujingjing@xtu.edu.cn

Supplementary Material

For each tensile specimen (five specimens per group), the fitted line for the initial linear region and the identified onset point of nonlinear deformation are provided in Figure S1.

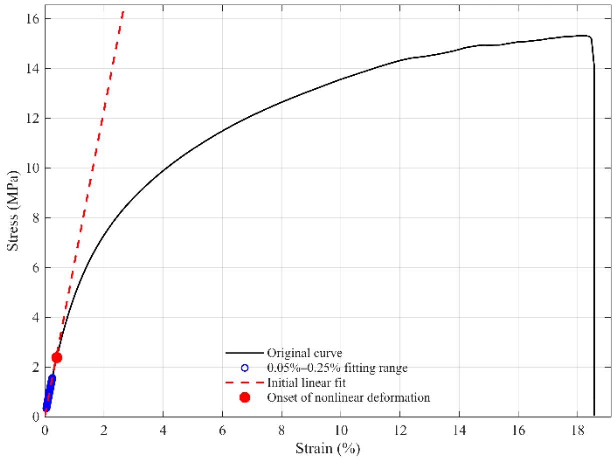

(a)-(1)

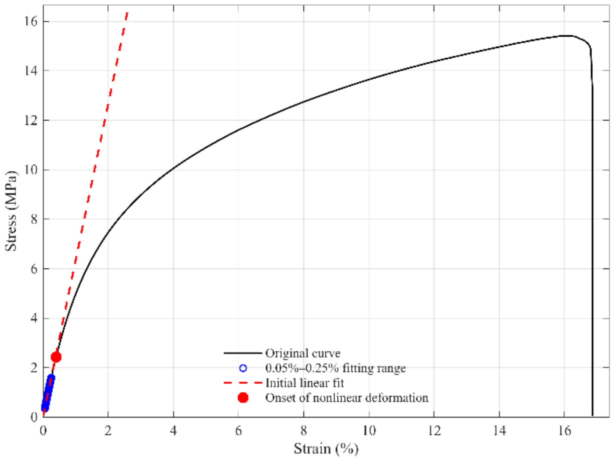

(a)-(2)

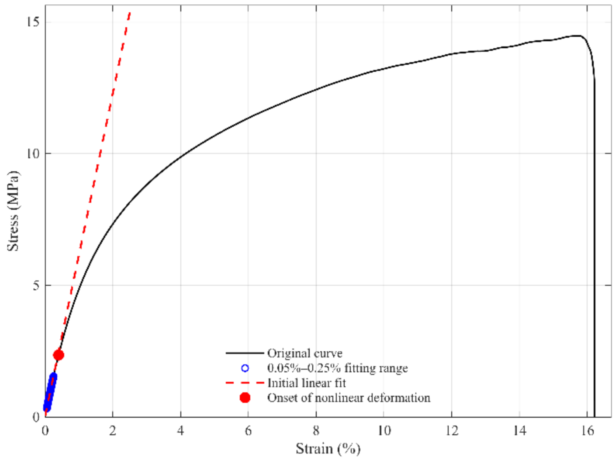
Stress (MPa)
Strain (%)
Original curve
0.05%–0.25% fitting range
Initial linear fit
Onset of nonlinear deformation

(a)-(3)

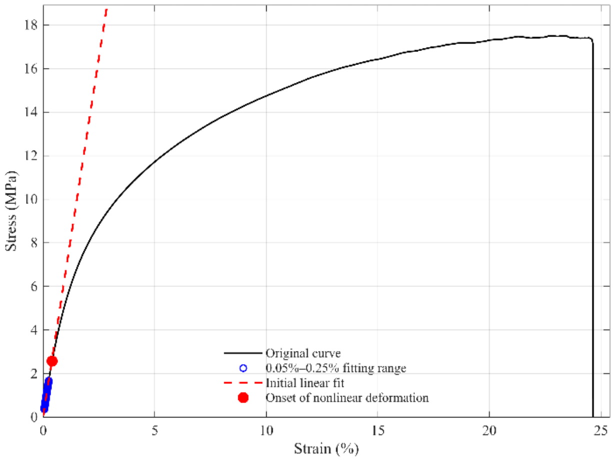
Stress (MPa)
Strain (%)
Original curve
0.05%–0.25% fitting range
Initial linear fit
Onset of nonlinear deformation

(a)-(3)

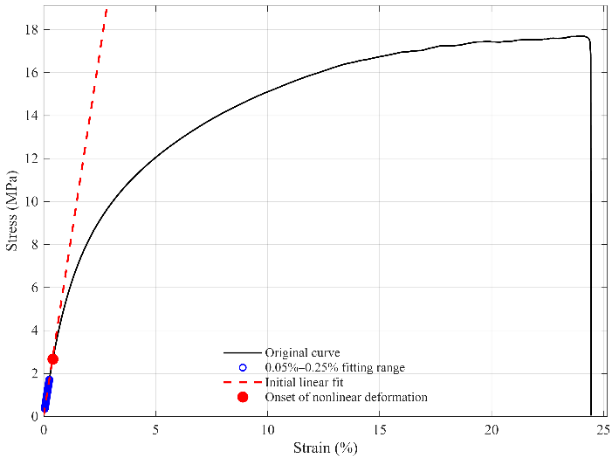
Stress (MPa)
Strain (%)
Original curve
0.05%–0.25% fitting range
Initial linear fit
Onset of nonlinear deformation

(a)-(5)

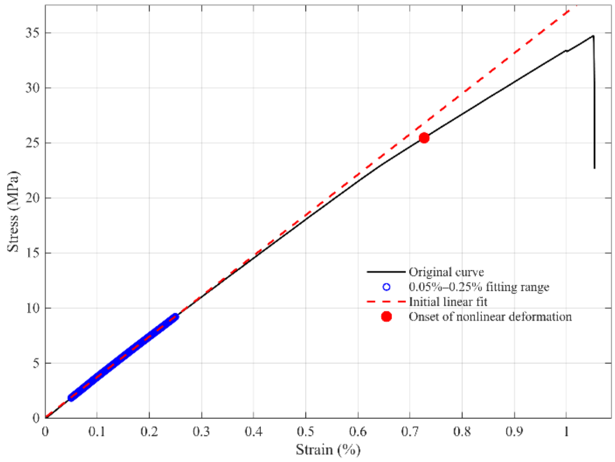
Stress (MPa)
Strain (%)
Original curve
0.05%–0.25% fitting range
Initial linear fit
Onset of nonlinear deformation

(b)-(1)

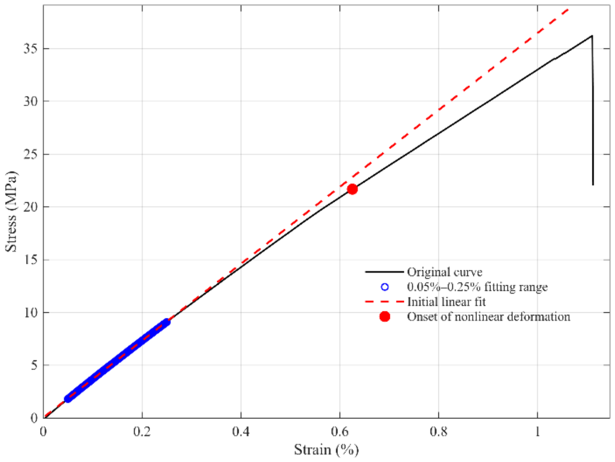
Stress (MPa)
Strain (%)
Original curve
0.05%–0.25% fitting range
Initial linear fit
Onset of nonlinear deformation

(b)-(2)

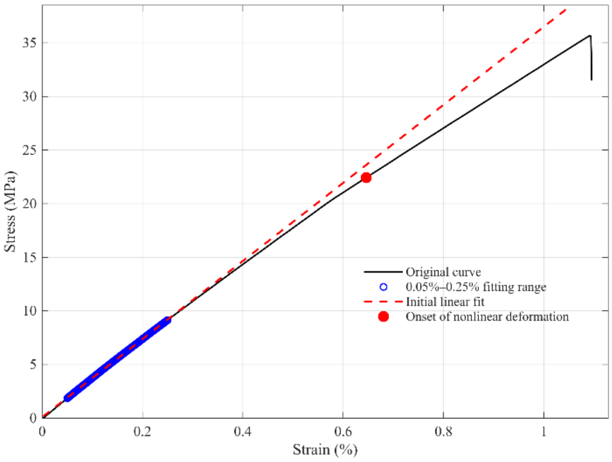
Stress (MPa)
Strain (%)
Original curve
0.05%–0.25% fitting range
Initial linear fit
Onset of nonlinear deformation

(b)-(3)

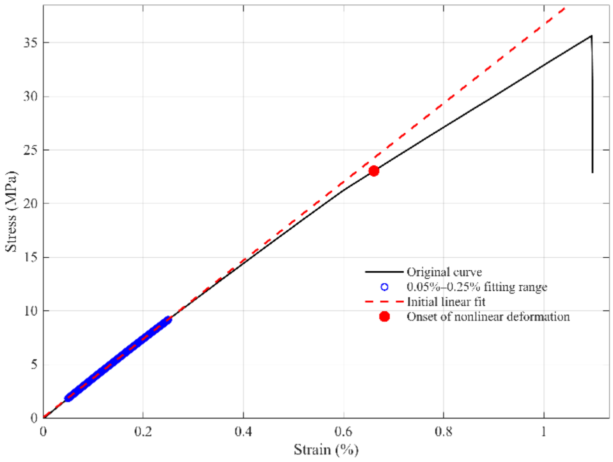
Stress (MPa)
Strain (%)
Original curve
0.05%–0.25% fitting range
Initial linear fit
Onset of nonlinear deformation

(b)-(4)

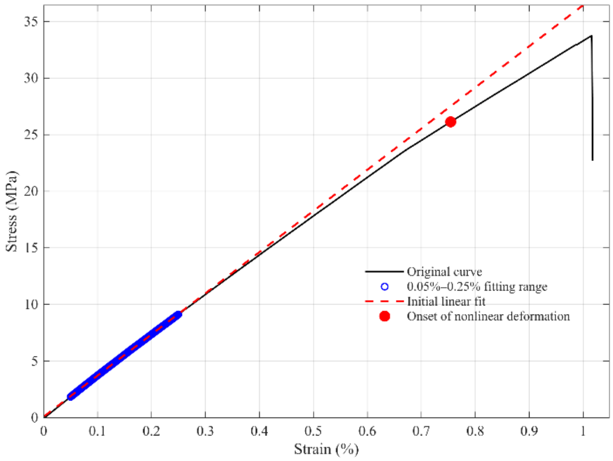
Stress (MPa)
Strain (%)
Original curve
0.05%–0.25% fitting range
Initial linear fit
Onset of nonlinear deformation

(b)-(5)

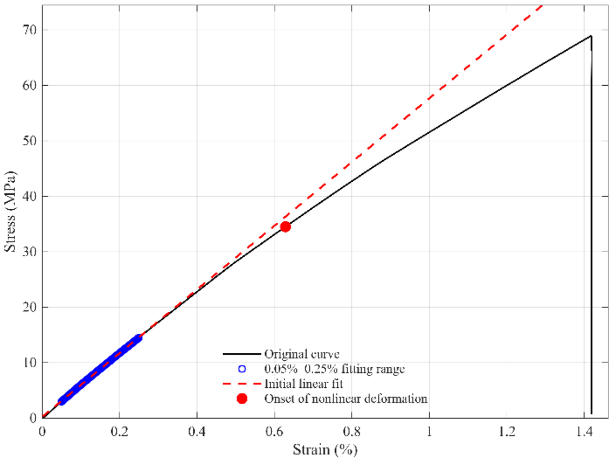
Stress (MPa)
Strain (%)
Original curve
0.05% 0.25% fitting range
Initial linear fit
Onset of nonlinear deformation

(c)-(1)

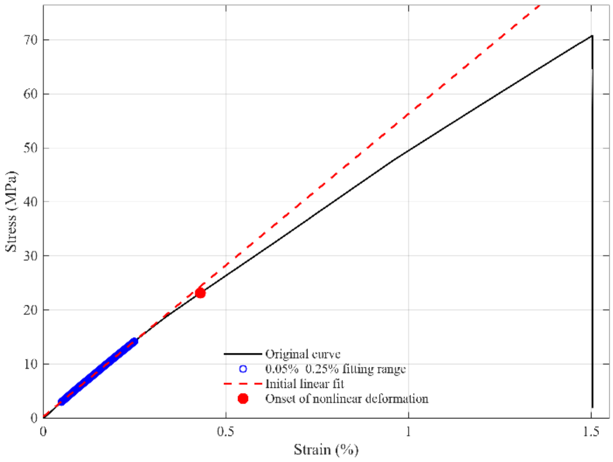
Stress (MPa)
Strain (%)
Original curve
0.05% 0.25% fitting range
Initial linear fit
Onset of nonlinear deformation

(c)-(2)

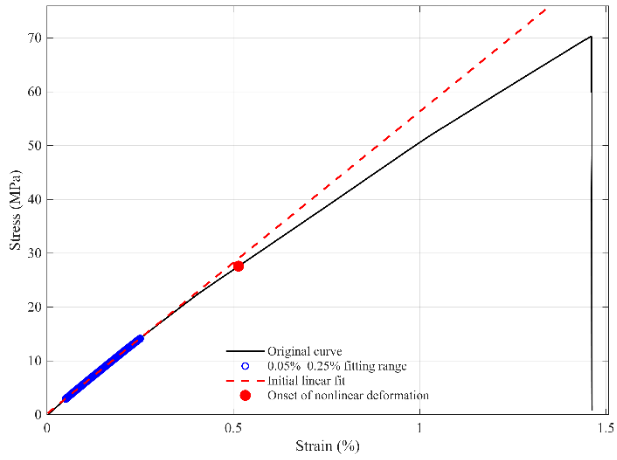
Stress (MPa)
Strain (%)
Original curve
0.05% 0.25% fitting range
Initial linear fit
Onset of nonlinear deformation

(c)-(3)

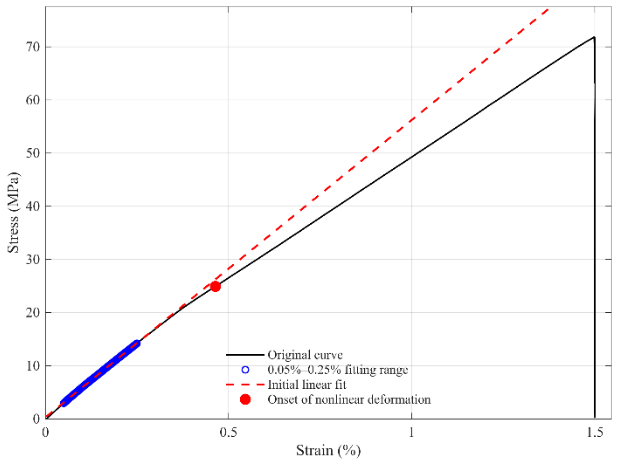
Stress (MPa)
Strain (%)
Original curve
0.05%–0.25% fitting range
Initial linear fit
Onset of nonlinear deformation

(c)-(3)

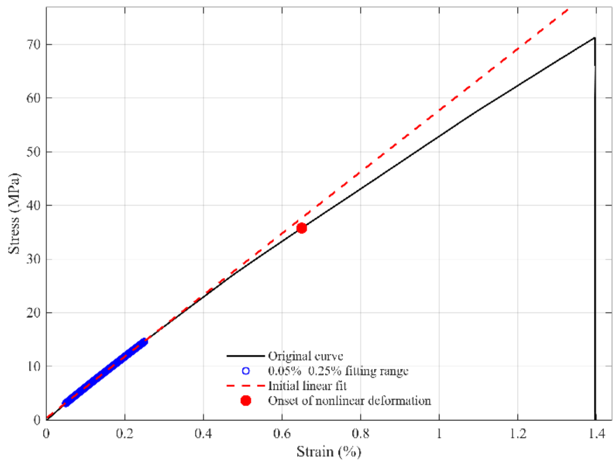
Stress (MPa)
Strain (%)
Original curve
0.05% 0.25% fitting range
Initial linear fit
Onset of nonlinear deformation

(c)-(5)

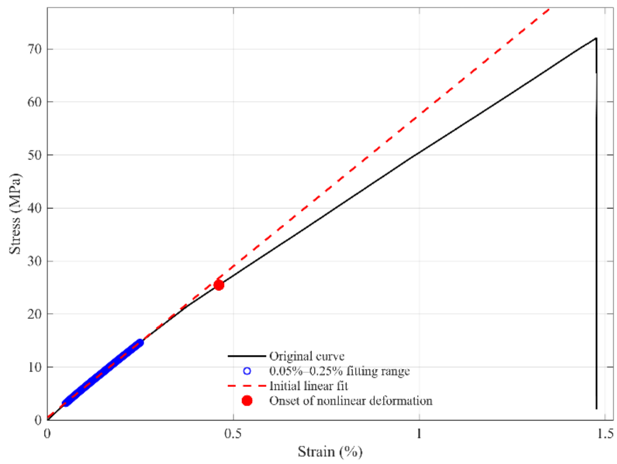
Stress (MPa)
Strain (%)
Original curve
0.05%–0.25% fitting range
Initial linear fit
Onset of nonlinear deformation

(d)-(1)

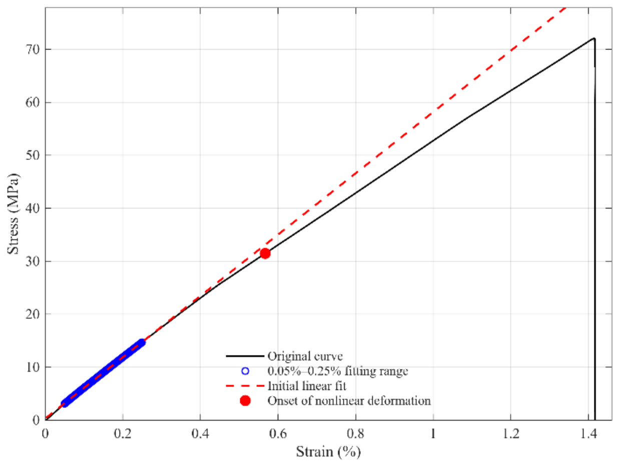
Stress (MPa)
Strain (%)
Original curve
0.05%–0.25% fitting range
Initial linear fit
Onset of nonlinear deformation

(d)-(2)

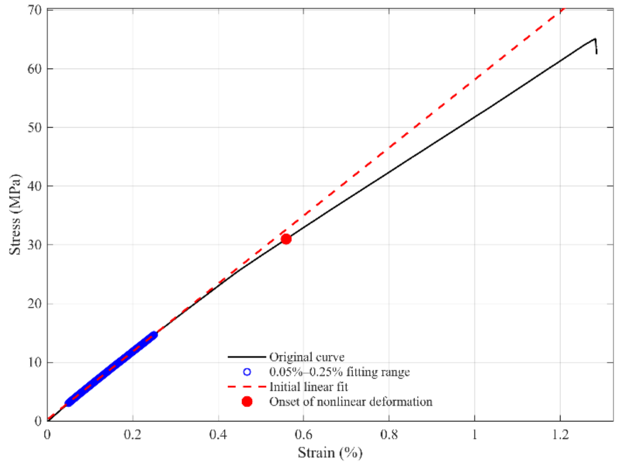
Stress (MPa)
Strain (%)
Original curve
0.05%–0.25% fitting range
Initial linear fit
Onset of nonlinear deformation
(d)-(3)

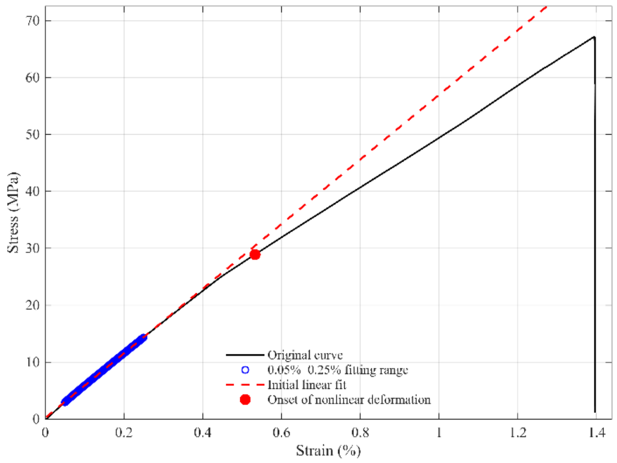
Stress (MPa)
Strain (%)
Original curve
0.05% 0.25% fitting range
Initial linear fit
Onset of nonlinear deformation
(d)-(4)

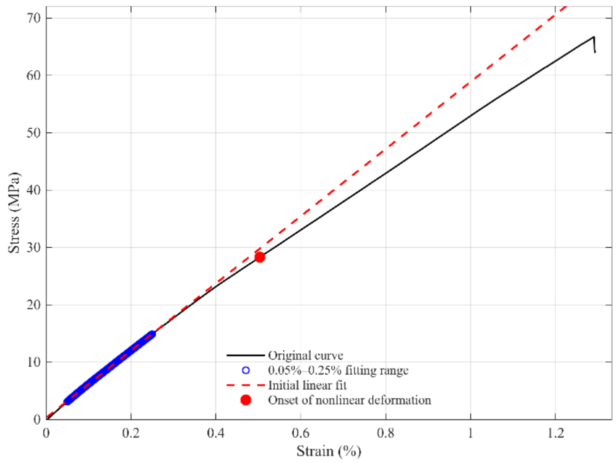
Stress (MPa)
Strain (%)
Original curve
0.05%–0.25% fitting range
Initial linear fit
Onset of nonlinear deformation
(d)-(5)

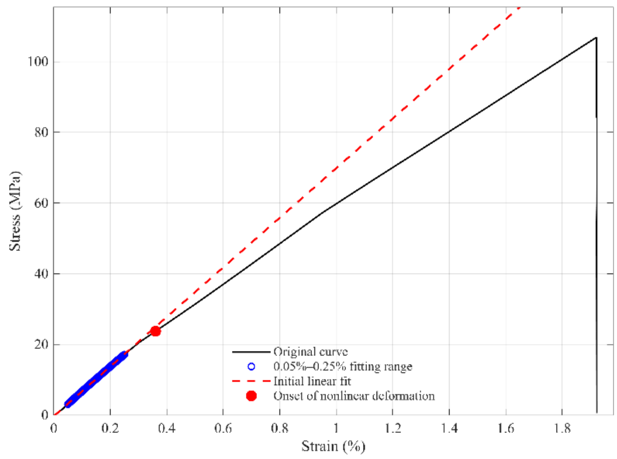
Stress (MPa)
Strain (%)
Original curve
0.05%–0.25% fitting range
Initial linear fit
Onset of nonlinear deformation
(e)-(1)

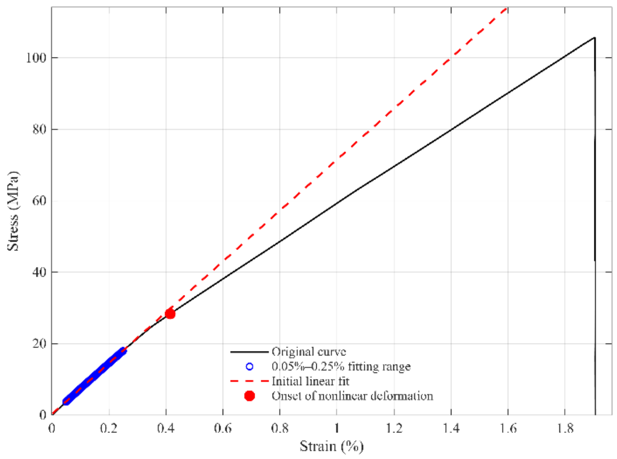
Stress (MPa)
Strain (%)
Original curve
0.05%–0.25% fitting range
Initial linear fit
Onset of nonlinear deformation
(e)-(2)

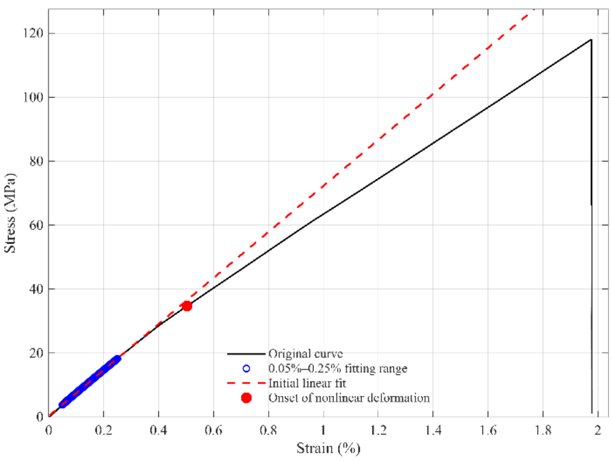

Stress (MPa)
Strain (%)
Original curve
0.05%–0.25% fitting range
Initial linear fit
Onset of nonlinear deformation

(e)-(3)

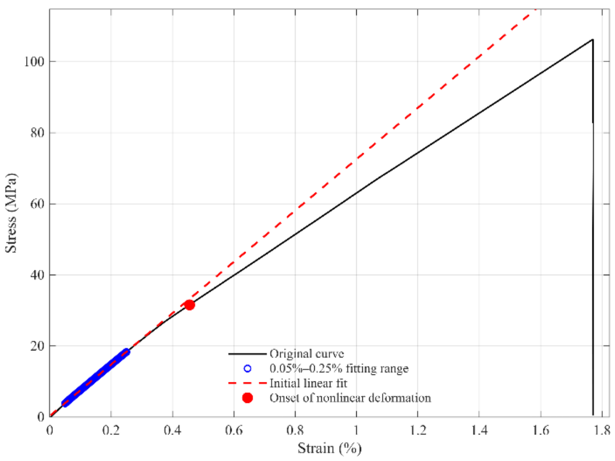

Stress (MPa)
Strain (%)
Original curve
0.05%–0.25% fitting range
Initial linear fit
Onset of nonlinear deformation

(e)-(3)

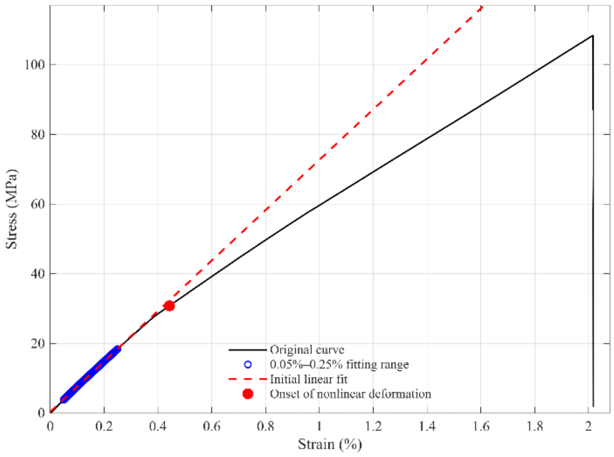

Stress (MPa)
Strain (%)
Original curve
0.05%–0.25% fitting range
Initial linear fit
Onset of nonlinear deformation

(e)-(5)

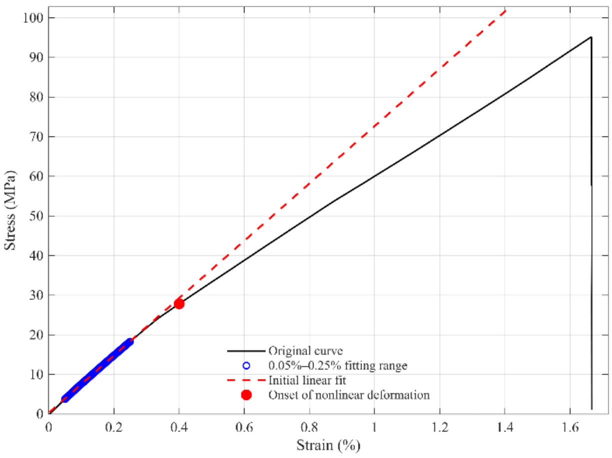

Stress (MPa)
Strain (%)
Original curve
0.05%–0.25% fitting range
Initial linear fit
Onset of nonlinear deformation

(f)-(1)

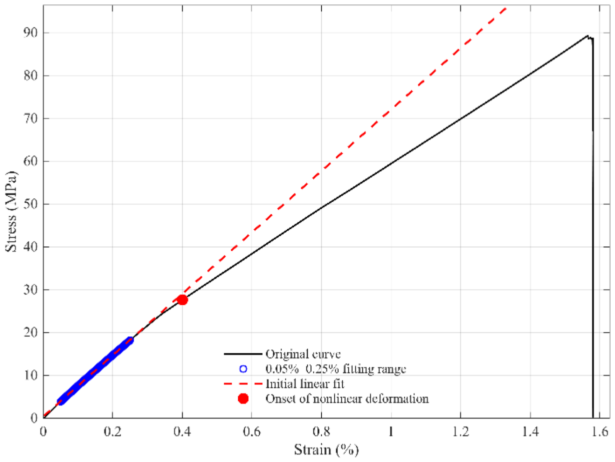

Stress (MPa)
Strain (%)
Original curve
0.05% 0.25% fitting range
Initial linear fit
Onset of nonlinear deformation

(f)-(2)

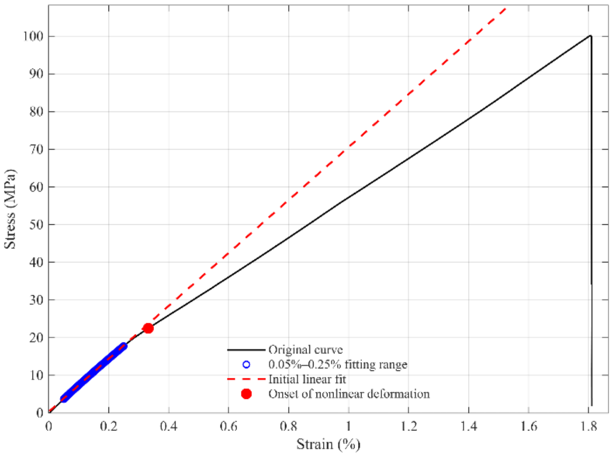

(f)-(3)

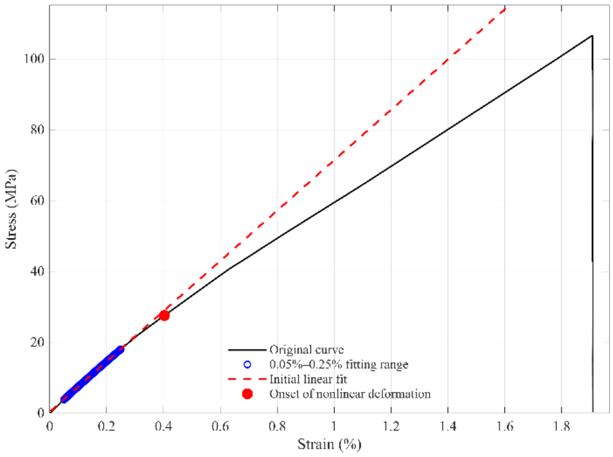

(f)-(4)

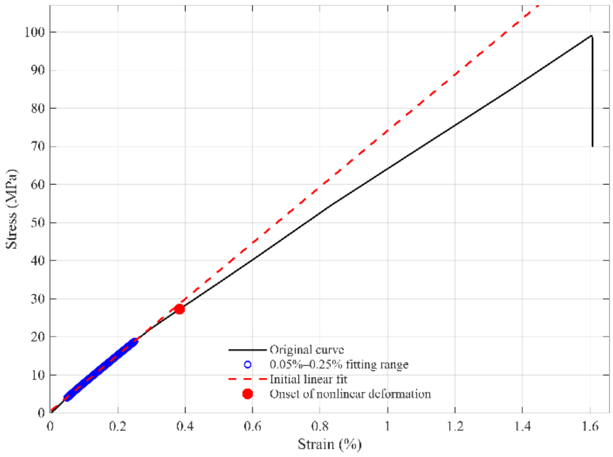

(f)-(5)

Figure S1. Fitted line for the initial linear region and onset point of nonlinear deformation of each tensile specimen: (a) G0; (b) G1; (c) G2C; (d) G2S; (e) G3C; (f) G3S.

The complete flexural stress–strain curves up to the complete failure in flexural load-carrying capacity of the specimens are provided in Figure S2.

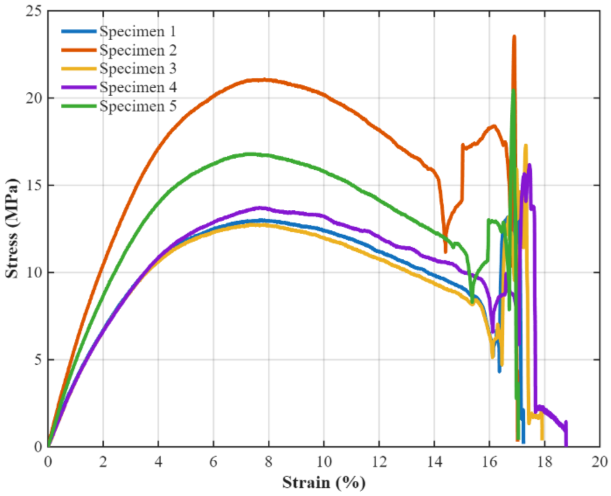

(a)

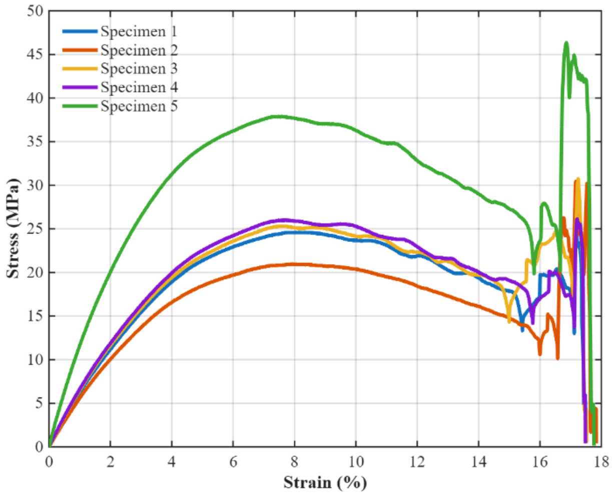

(b)

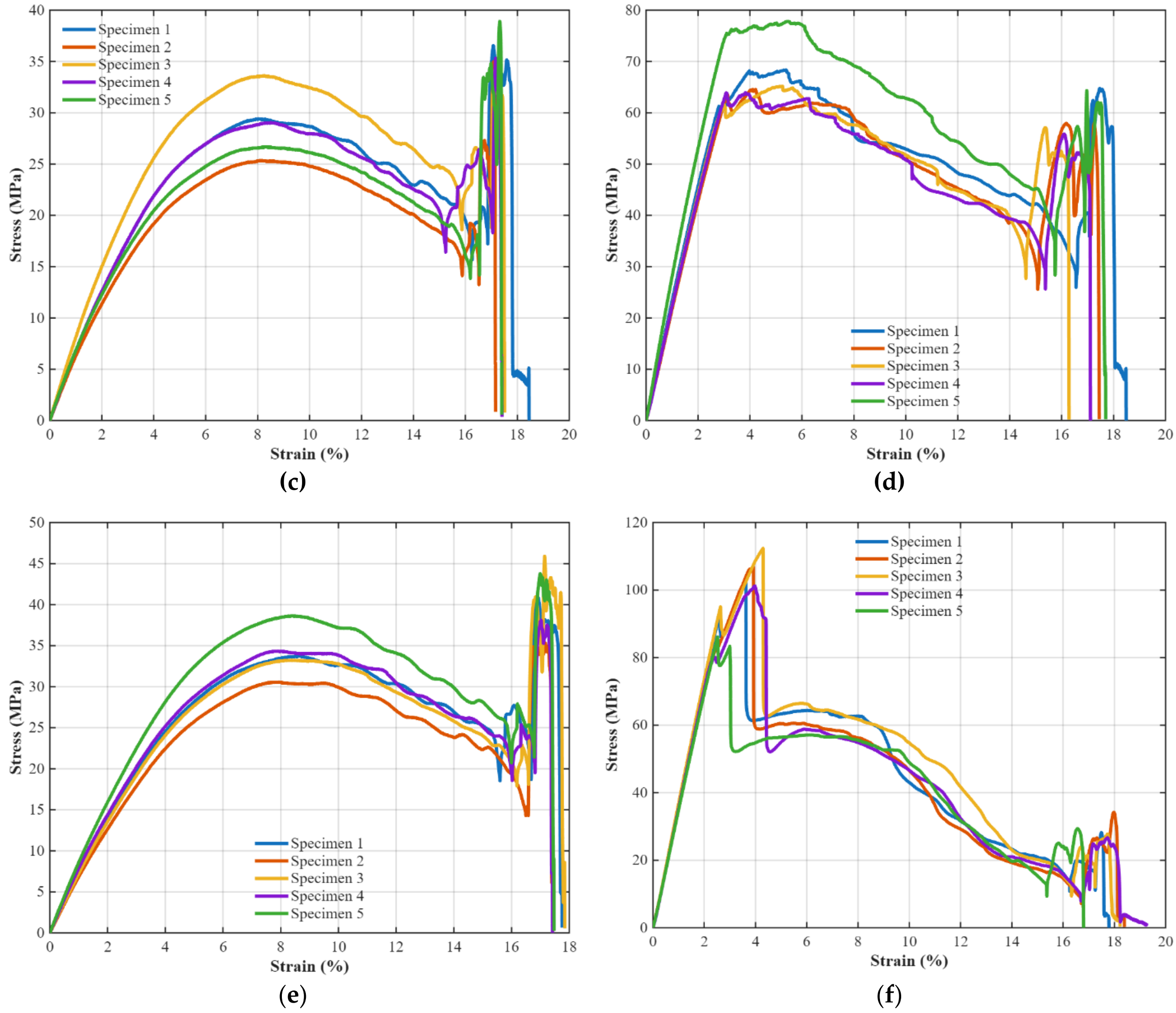

Figure S2. Complete flexural stress–strain curves of flexural specimens: (a) G0; (b) G1; (c) G2C; (d) G2S; (e) G3C; (f) G3S.

For each flexural specimen (five specimens per group), the fitted line for the initial linear region and the identified onset point of nonlinear deformation are provided in Figure S3.

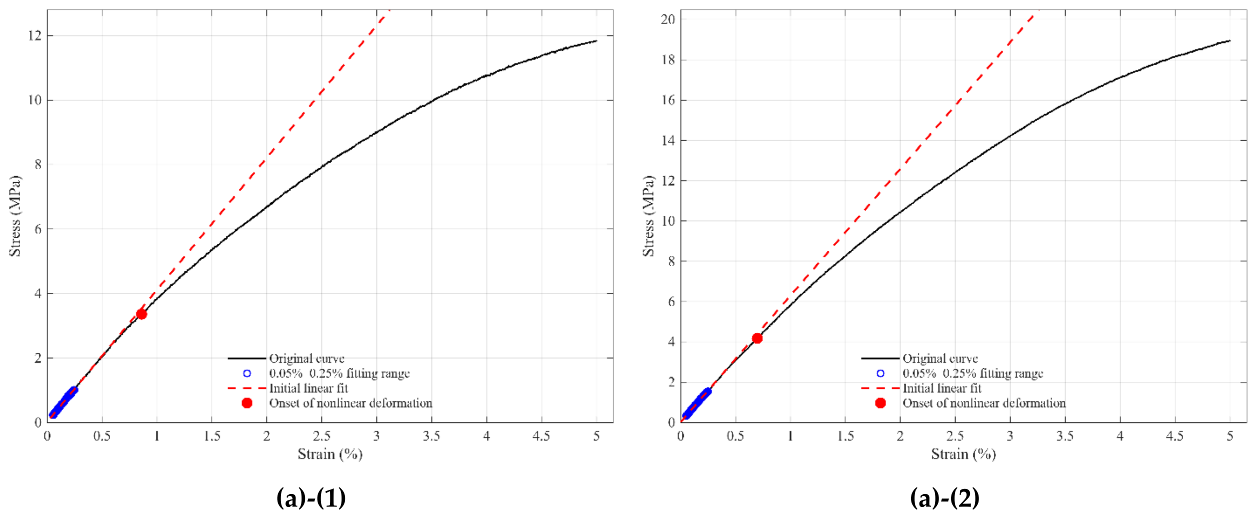

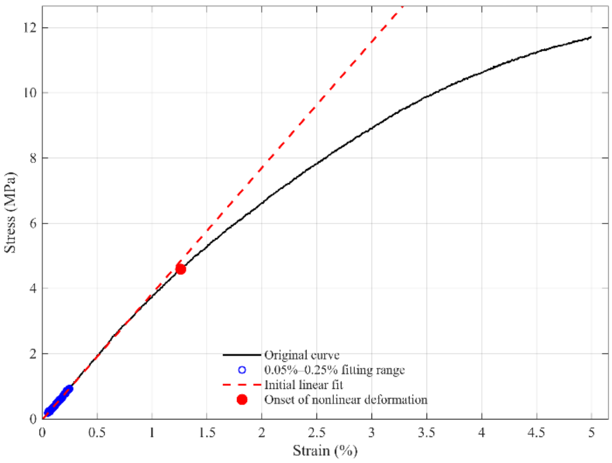

Stress (MPa)
Strain (%)
Original curve
0.05%–0.25% fitting range
Initial linear fit
Onset of nonlinear deformation

(a)-(3)

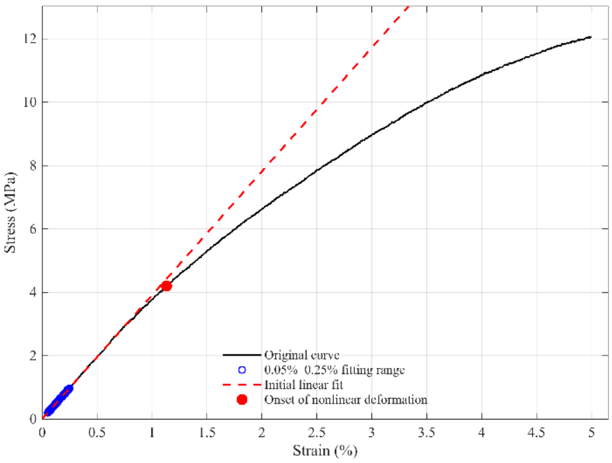

Stress (MPa)
Strain (%)
Original curve
0.05% 0.25% fitting range
Initial linear fit
Onset of nonlinear deformation

(a)-(3)

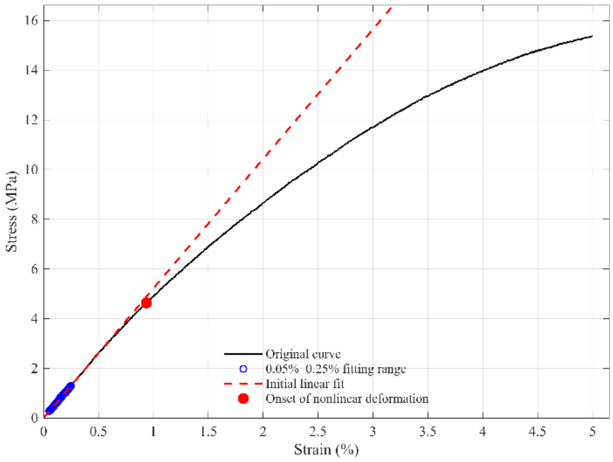

Stress (MPa)
Strain (%)
Original curve
0.05% 0.25% fitting range
Initial linear fit
Onset of nonlinear deformation

(a)-(5)

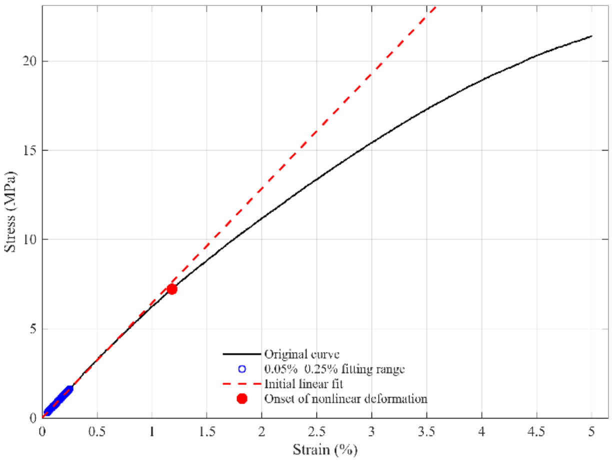

Stress (MPa)
Strain (%)
Original curve
0.05% 0.25% fitting range
Initial linear fit
Onset of nonlinear deformation

(b)-(1)

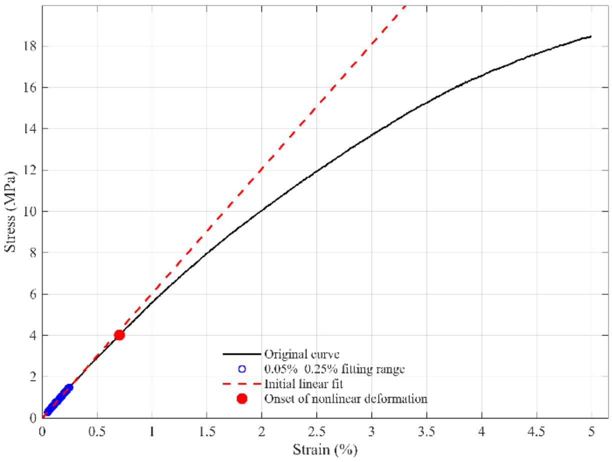

Stress (MPa)
Strain (%)
Original curve
0.05% 0.25% fitting range
Initial linear fit
Onset of nonlinear deformation

(b)-(2)

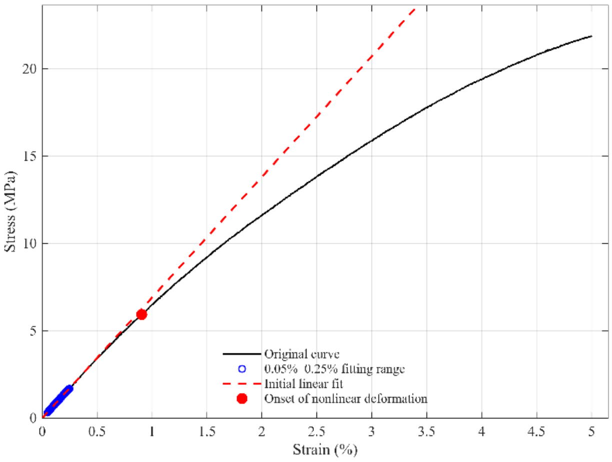
Stress (MPa)
Strain (%)
Original curve
0.05% 0.25% fitting range
Initial linear fit
Onset of nonlinear deformation

(b)-(3)

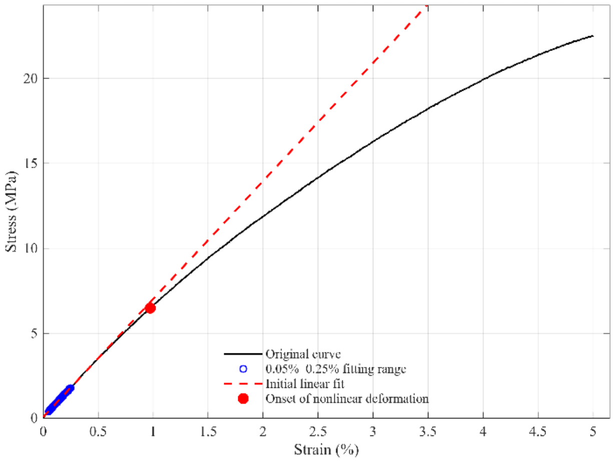
Stress (MPa)
Strain (%)
Original curve
0.05% 0.25% fitting range
Initial linear fit
Onset of nonlinear deformation

(b)-(4)

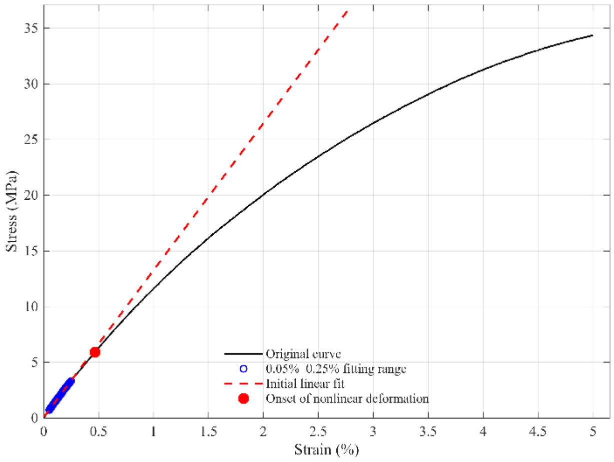
Stress (MPa)
Strain (%)
Original curve
0.05% 0.25% fitting range
Initial linear fit
Onset of nonlinear deformation

(b)-(5)

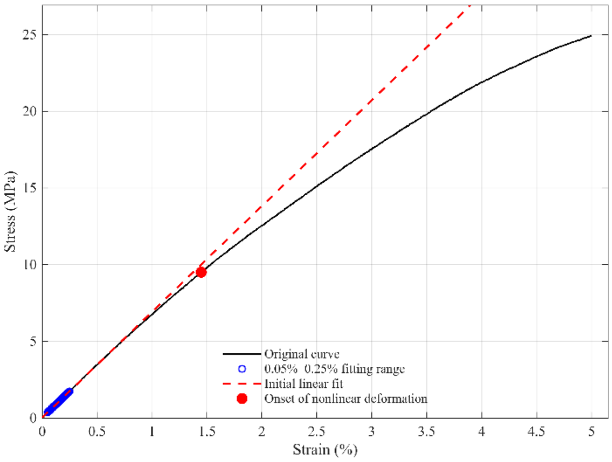
Stress (MPa)
Strain (%)
Original curve
0.05% 0.25% fitting range
Initial linear fit
Onset of nonlinear deformation

(c)-(1)

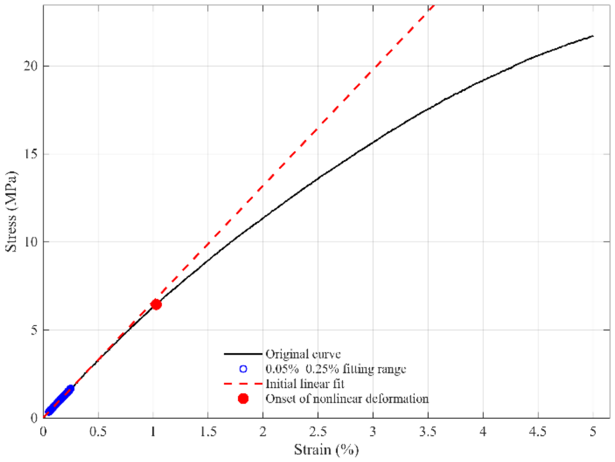
Stress (MPa)
Strain (%)
Original curve
0.05% 0.25% fitting range
Initial linear fit
Onset of nonlinear deformation

(c)-(2)

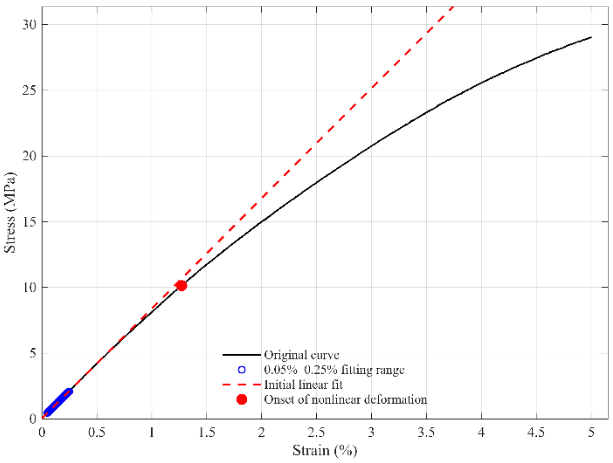
Stress (MPa)
Strain (%)
Original curve
0.05% 0.25% fitting range
Initial linear fit
Onset of nonlinear deformation

(c)-(3)

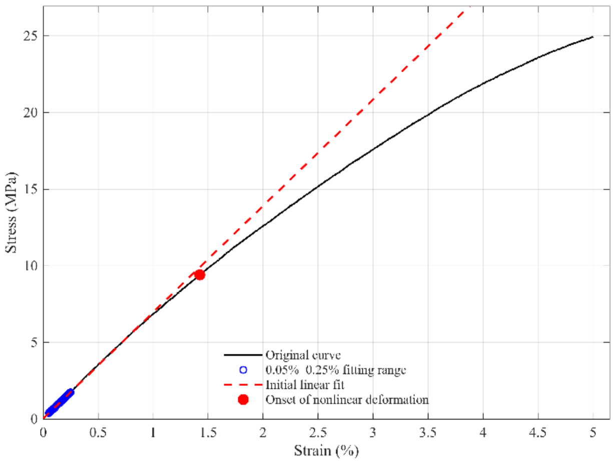
Stress (MPa)
Strain (%)
Original curve
0.05% 0.25% fitting range
Initial linear fit
Onset of nonlinear deformation

(c)-(3)

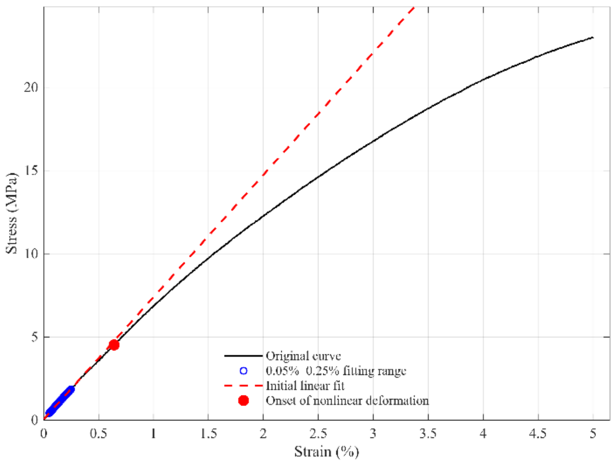
Stress (MPa)
Strain (%)
Original curve
0.05% 0.25% fitting range
Initial linear fit
Onset of nonlinear deformation

(c)-(5)

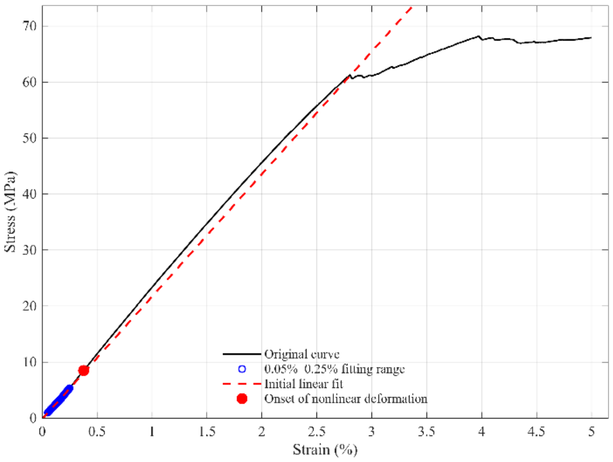
Stress (MPa)
Strain (%)
Original curve
0.05% 0.25% fitting range
Initial linear fit
Onset of nonlinear deformation

(d)-(1)

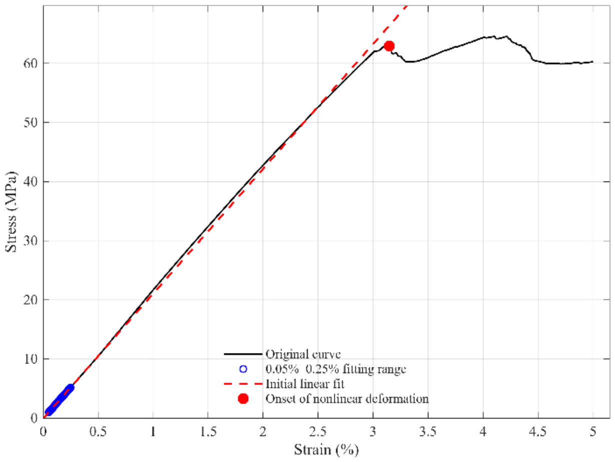
Stress (MPa)
Strain (%)
Original curve
0.05% 0.25% fitting range
Initial linear fit
Onset of nonlinear deformation

(d)-(2)

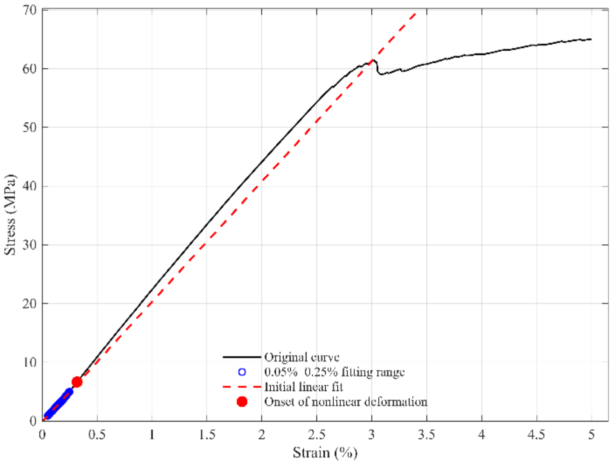
Stress (MPa)
Strain (%)
Original curve
0.05% 0.25% fitting range
Initial linear fit
Onset of nonlinear deformation

(d)-(3)

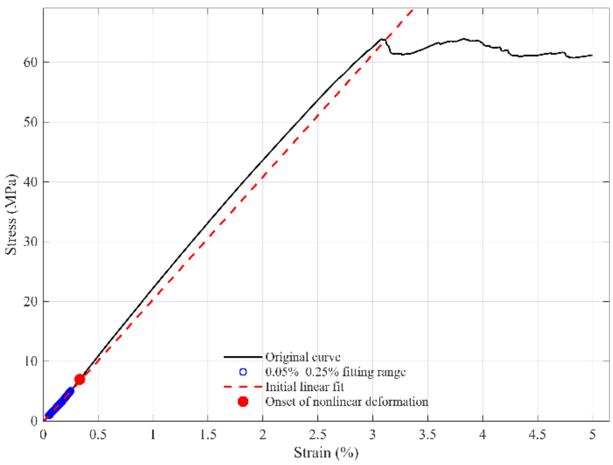
Stress (MPa)
Strain (%)
Original curve
0.05% 0.25% fitting range
Initial linear fit
Onset of nonlinear deformation

(d)-(4)

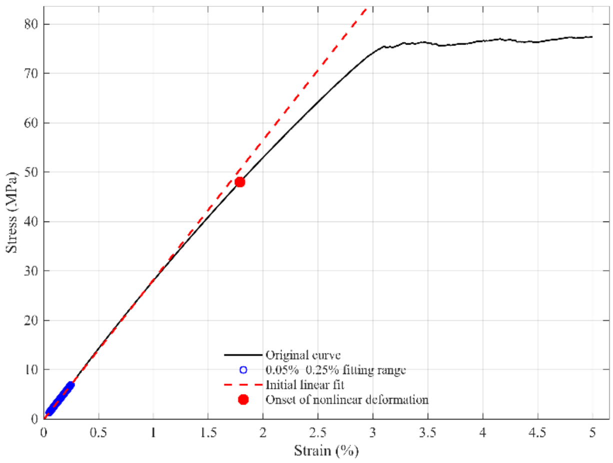
Stress (MPa)
Strain (%)
Original curve
0.05% 0.25% fitting range
Initial linear fit
Onset of nonlinear deformation

(d)-(5)

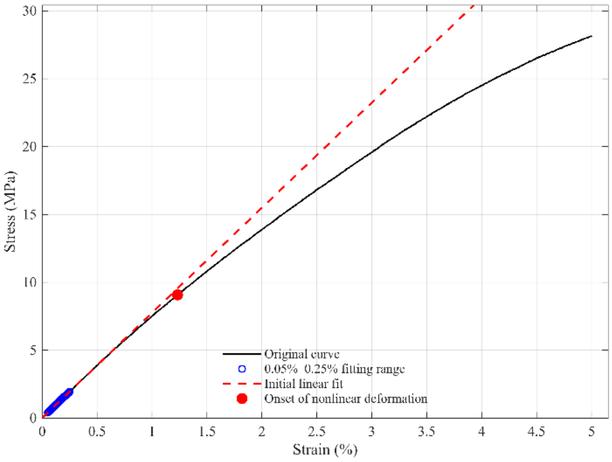
Stress (MPa)
Strain (%)
Original curve
0.05% 0.25% fitting range
Initial linear fit
Onset of nonlinear deformation

(e)-(1)

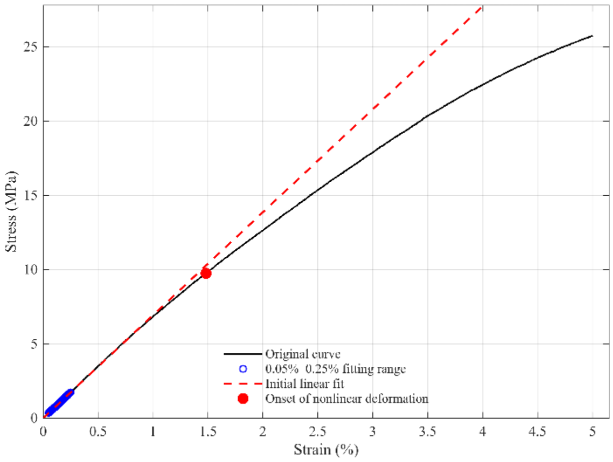
Stress (MPa)
Strain (%)
Original curve
0.05% 0.25% fitting range
Initial linear fit
Onset of nonlinear deformation

(e)-(2)

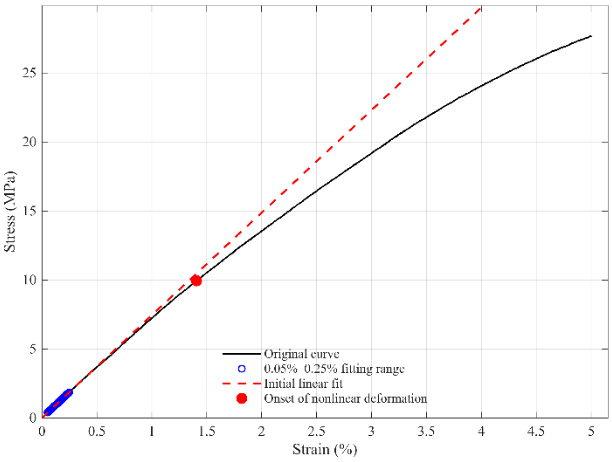
Stress (MPa)
Strain (%)
Original curve
0.05% 0.25% fitting range
Initial linear fit
Onset of nonlinear deformation

(e)-(3)

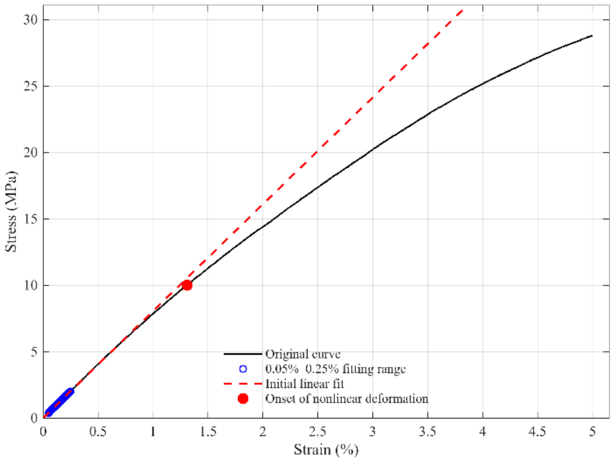
Stress (MPa)
Strain (%)
Original curve
0.05% 0.25% fitting range
Initial linear fit
Onset of nonlinear deformation

(e)-(3)

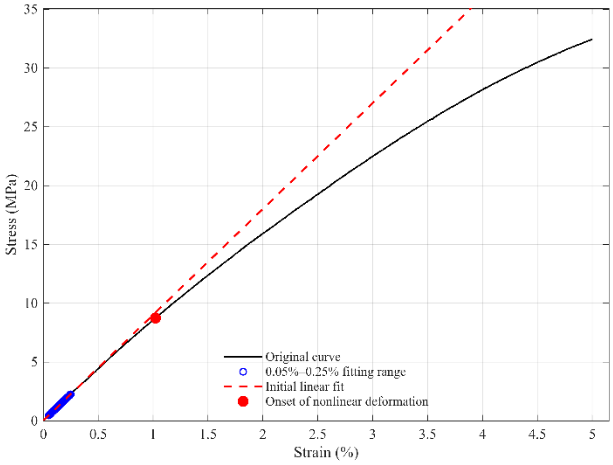
Stress (MPa)
Strain (%)
Original curve
0.05%–0.25% fitting range
Initial linear fit
Onset of nonlinear deformation

(e)-(5)

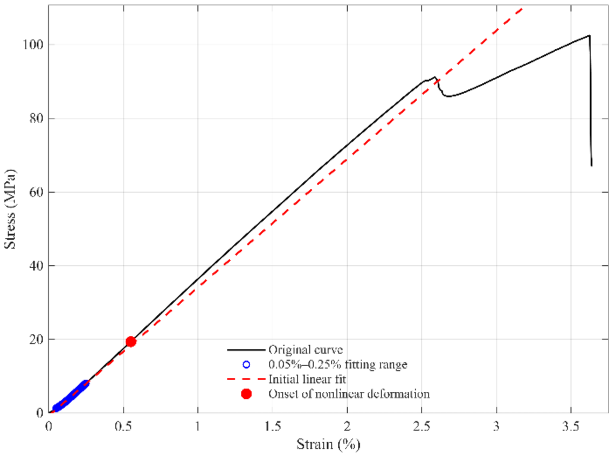
Stress (MPa)
Strain (%)
Original curve
0.05%–0.25% fitting range
Initial linear fit
Onset of nonlinear deformation

(f)-(1)

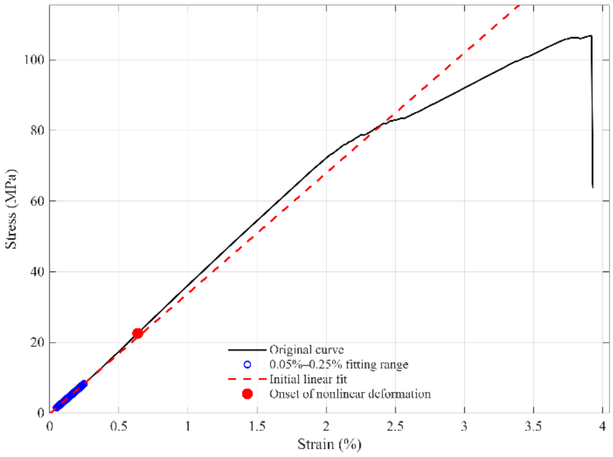
Stress (MPa)
Strain (%)
Original curve
0.05%–0.25% fitting range
Initial linear fit
Onset of nonlinear deformation

(f)-(2)

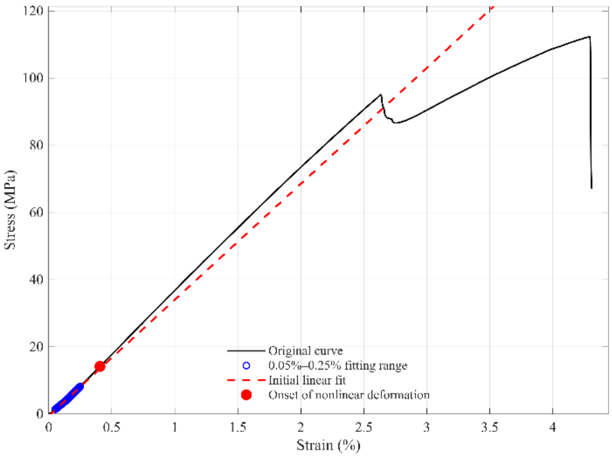

(f)-(3)

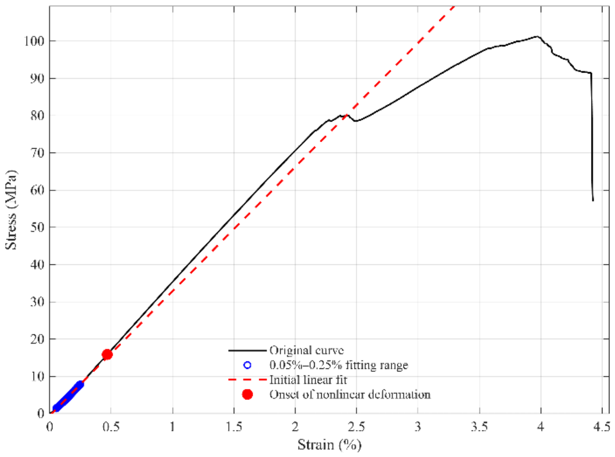

(f)-(4)

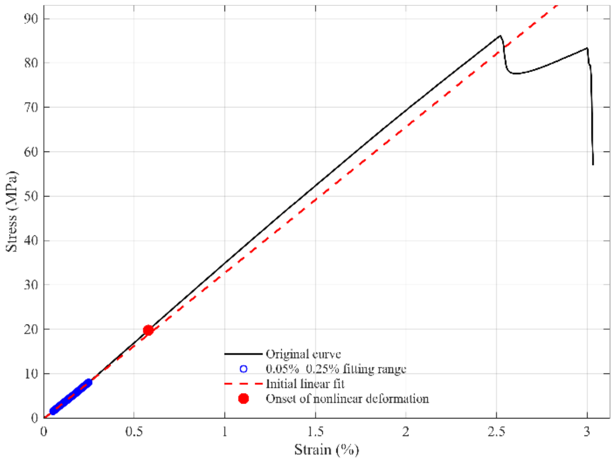

(f)-(5)

Figure S3. Fitted line for the initial linear region and onset point of nonlinear deformation of each flexural specimen: (a) G0; (b) G1; (c) G2C; (d) G2S; (e) G3C; (f) G3S.
